# Factors Influencing Self‐Reported Health in Puerto Rican Populations: A Systematic Review

**DOI:** 10.1002/hsr2.73002

**Published:** 2026-08-09

**Authors:** Anthony Vincent Razzano

**Affiliations:** ^1^ Department of Public Health, Zuckerberg College of Health Sciences University of Massachusetts Lowell Lowell Massachusetts USA

**Keywords:** health belief model, health disparities, health‐related quality of life, Puerto Rican, self‐rated health, self‐reported health, systematic review

## Abstract

**Background and Aims:**

Puerto Ricans experience disproportionate chronic disease burden, structural barriers to care, and disparities in health outcomes. Self‐reported health is a validated indicator of morbidity, mortality, and healthcare utilization, yet its determinants in Puerto Rican populations remain insufficiently synthesized. This review examined factors associated with self‐reported health among Puerto Rican populations in mainland United States and island settings.

**Methods:**

A systematic review was conducted following Preferred Reporting Items for Systematic Reviews and Meta‐Analyses guidelines. Nine databases were searched for English‐language studies published from January 1, 1996, through January 31, 2026, using Puerto Rican population terms combined with self‐reported health, self‐rated health, subjective health, general health status, health perception, and health‐related quality‐of‐life terms. Eligible studies reported original empirical data on self‐reported health or health‐related quality of life as a primary or secondary outcome in Puerto Rican samples. Study quality was appraised using Joanna Briggs Institute checklists. The Health Belief Model was applied post hoc to organize extracted findings.

**Results:**

Of 2059 records identified, 1271 remained after deduplication; 38 reports underwent full‐text review, and 33 studies were included in qualitative synthesis. Most studies were cross‐sectional or quasi‐experimental, with four cohort designs. Chronic disease burden, functional limitation, depressive symptoms, and allostatic load were consistently associated with poorer self‐reported health. Socioeconomic disadvantage, language barriers, discrimination, psychosocial stress, and provider knowledge gaps further shaped outcomes. Acculturation influenced health perceptions and introduced measurement bias, with less acculturated individuals tending to underreport poor health. Nativity effects persisted across study designs, with island birth associated with durable health disadvantages independent of current residence. Aggregating Puerto Ricans into pan‐Hispanic categories obscured population‐specific patterns.

**Conclusion:**

Poor self‐reported health among Puerto Rican populations reflects intersecting chronic disease, socioeconomic disadvantage, acculturation‐related measurement bias, and nativity. Disaggregated data, culturally validated measures, longitudinal designs, and provider education on Puerto Rican health contexts are needed to improve research accuracy and quality of care.

## 1 Introduction

Self‐reported health is a widely used indicator of how individuals perceive their own health status, and it consistently predicts hospitalizations, emergency department use, morbidity, and mortality across populations [1]. Because a single self‐rating item captures a synthesis of biological, psychological, and social experience, understanding which populations self‐rate their health, and how, is as important as the responses themselves: self‐reported health can reveal disparities in access to care and representation in health research that are not always visible in clinical measures alone.

Puerto Ricans have historically faced systemic barriers to receiving health care. High poverty levels, fragile infrastructure, weakened public systems, and hazardous environmental conditions on the island collectively contribute to adverse health outcomes across individual, community, and population levels [2]. Puerto Ricans experience poorer health status and a higher prevalence of multiple chronic conditions than non‐Hispanic Whites and other Hispanic subgroups, reflecting substantial heterogeneity in health outcomes across Hispanic populations that can be obscured when these groups are analyzed as a single category [3].

This systematic review synthesizes evidence on the factors associated with self‐reported health within Puerto Rican populations, addressing a literature that remains fragmented across disease‐specific, disaster‐related, and general‐population studies. Clear research questions guide the evidence‐based healthcare process and link each review approach to specific inclusion criteria through a unified typology [4]. Adhering to Preferred Reporting Items for Systematic Reviews and Meta‐Analyses guidelines ensures a rigorous standard for identifying, selecting, and appraising literature [5]. The Population, Exposure, and Outcome framework refines study selection: among Puerto Rican populations (P), what factors (E) are associated with self‐reported health or related health‐status outcomes (O)? Following full‐text data extraction, the Health Belief Model was applied as an organizing interpretive framework, consistent with precedent for applying the model as an explanatory lens for health perceptions and behaviors [6], rather than as a criterion that determined which studies were eligible for inclusion. Its constructs, perceived susceptibility, severity, benefits, barriers, self‐efficacy, and cues to action, are used in Section Conclusion, 4 to structure the narrative synthesis of how individual health beliefs interact with broader social and structural influences.

## 2 Methods

### 2.1 Search Strategy

This systematic review is registered with the Open Science Framework (https://doi.org/10.17605/OSF.IO/E4U2Q) and prioritizes transparency and reproducibility. Ethical approval was not required for this systematic review, as it analyzes previously published data and does not involve direct human participants. The sole author and reviewer leveraged the University of Massachusetts Lowell library search engine access via the EBSCO platform to search nine databases: MEDLINE, Academic Search Ultimate, CINAHL, APA PsycInfo, Education Research Complete, Health and Psychosocial Instruments, Political Science Complete, America: History & Life, and ERIC. Search parameters restricted results to English‐language publications from January 1, 1996 to January 31, 2026. The search combined population terms (“Puerto Rican” OR “Puerto Ricans” OR “Puerto Rico” OR Boricua OR Boricuas) with a broadened set of outcome terms capturing self‐rated, self‐reported, self‐perceived, and subjective health, general health status, health perception(s), and health‐related quality of life. The complete search strategy is presented in Table 1. Applying this strategy returned 2059 records across the nine databases.

**Table 1:** The search strategy of the research.

Component	Details
Databases	MEDLINE (*n* = 966), Academic Search Ultimate (*n* = 460), CINAHL (*n* = 298), APA PsycInfo (*n* = 267), Education Research Complete (*n* = 45), Health and Psychosocial Instruments (*n* = 10), Political Science Complete (*n* = 6), America: History & Life (*n* = 4), ERIC (*n* = 3) (via EBSCO, University of Massachusetts Lowell library; nine databases searched in full, not limited by institutional full‐text access)
Date limits	January 1, 1996 to January 31, 2026
Language	English only
Study types	Original empirical research; cross‐sectional, quasi‐experimental, and cohort designs
Strategy	#1 AND #2
#1 Population	(“Puerto Rican” OR “Puerto Ricans” OR “Puerto Rico” OR Boricua OR Boricuas)
#2 Outcome	(“self‐rated health” OR “self rated health” OR “self‐reported health” OR “self reported health” OR “self‐perceived health”OR “self perceived health” OR “perceived health” OR “perceived health status” OR “subjective health” OR “subjective health status”OR “general health” OR “general health status” OR “health status” OR “health perception” OR “health perceptions” OR“health‐related quality of life” OR “health related quality of life” OR HRQoL OR “quality of life” OR “global health”)
Initial records	2059 records identified across nine databases
Deduplication	788 duplicate records removed via a three‐stage process (704 by DOI match, 82 by title match, 2 by abstract match); 1271 unique records remained for title/abstract screening
Included studies	33 studies meeting full eligibility criteria, retained for qualitative synthesis (from 38 full‐text reports assessed; 5 excluded)

### 2.2 Study Selection and Eligibility Criteria

**Figure 1:**
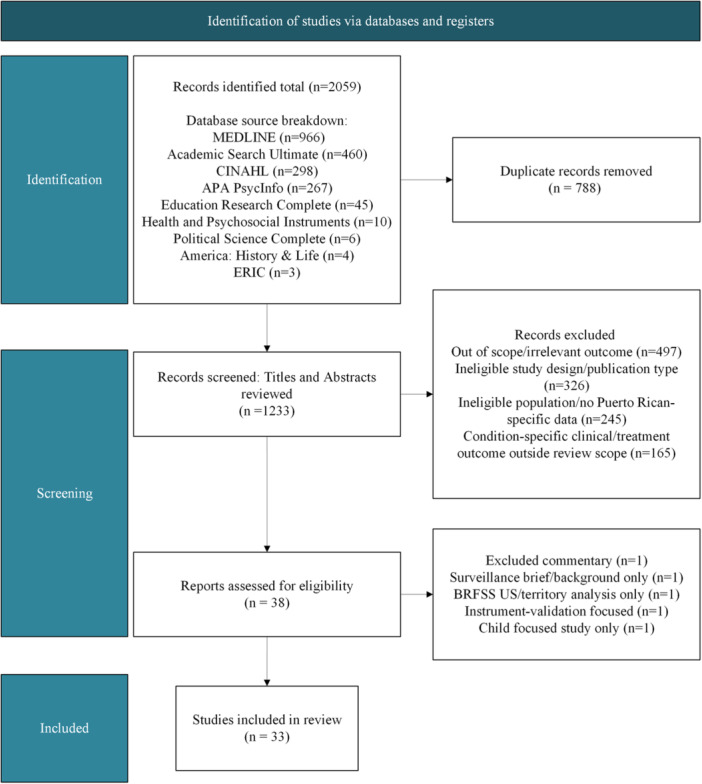
Preferred reporting items for systematic reviews and meta‐analyses flow diagram.

A three‐stage deduplication process, matching first on DOI, then on title, then on abstract, removed 788 duplicate records, leaving 1271 unique records for title and abstract screening. Screening excluded 1233 records: out of scope or irrelevant outcome (*n* = 497), ineligible study design or publication type (*n* = 326), ineligible population or no Puerto Rican‐specific data (*n* = 245), and condition‐specific clinical or treatment outcomes outside the review's scope (*n* = 165). The remaining 38 reports were assessed for full‐text eligibility; 5 were excluded (a rights‐based commentary that did not report original empirical analysis of self‐rated health or health‐related quality of life determinants, *n* = 1; a legacy national surveillance brief retained as background context in Section 5 but not eligible for full‐text synthesis alongside more recent, population‐representative studies, *n* = 1; a broader multi‐territory analysis in which Puerto Rico could not be extracted as a distinct analytic subgroup, *n* = 1; an instrument‐validation study focused on quality‐of‐life scale psychometrics rather than determinants of self‐rated health or health status, *n* = 1; and a pediatric instrument‐cultural‐adaptation study outside the adult population scope of this review, *n* = 1), yielding 33 studies for qualitative synthesis. Eligible studies reported original empirical data on self‐reported health or health‐related quality of life as a primary or secondary outcome in Puerto Rican samples, in the mainland United States or island Puerto Rico. Geographic requirements focused on the mainland United States and the island of Puerto Rico, though one comparative study reporting a distinct Puerto Rico subgroup alongside other countries was retained given its direct relevance to an understudied population (Section 4.10). Grey literature sources such as dissertations, conference proceedings, and government or institutional reports were not systematically searched; this scope decision is addressed as a limitation in Section 5. The Preferred Reporting Items for Systematic Reviews and Meta‐Analyses flowchart is presented in Figure 1 to visualize the study selection and exclusion process. Table 2 summarizes study characteristics, statistical findings, and Health Belief Model constructs for each included study.

**Table 2:** Summary of included studies (*N* = 33).

Study information	Scope	Type of study	Population	Statistical relevance and key findings	Health belief model construct(s) identified
Jaen et al. (1998) [7] Patterns of Dysphoria in a Puerto Rican Urban Community	United States Urban mainland community	Cross‐sectional community survey; chi‐squared analysis, Spearman correlation, and linear regression	704 adults from a cross‐sectional sample in a poor, predominantly Puerto Rican urban community	Twenty percent of respondents were dysphoric. Perceived distance to hospitals and language barriers with providers were associated with significantly higher likelihood of dysphoria. Age, gender, and health status were associated with dysphoria after adjustment for other variables.	Perceived Barriers; Perceived Susceptibility
Rao et al. (2007) [8] The Health Related Quality of Life Outcomes of English and Spanish Speaking Persons Living with HIV/AIDS from the Continental United States and Puerto Rico	United States mainland and Puerto Rico (island)	Cross‐sectional; Functional Assessment of HIV Infection quality‐of‐life outcomes; comparison by language of administration and site	Adults living with HIV/AIDS recruited from continental United States and Puerto Rico HIV clinical sites, compared by English‐ versus Spanish‐language survey administration.	Health‐related quality of life differed by language of survey administration and clinical site, with Spanish‐speaking participants from Puerto Rico reporting a distinct symptom and well‐being profile relative to English‐speaking mainland participants. Findings supported the need for language‐ and culture‐concordant HRQoL assessment in HIV care.	Perceived Susceptibility; Perceived Severity; Perceived Barriers
Castaneda‐Sceppa et al. (2010) [9] Physical Function and Health Status in Aging Puerto Rican Adults	United States Boston, MA (mainland)	Cross‐sectional; baseline data from Boston Puerto Rican Health Study	1,357 Puerto Rican adults aged 45–75 in the greater Boston area	Older women aged 60–75 had the poorest physical function. Obesity, diabetes, depression, history of heart attack, stroke, and arthritis were each associated with poor physical function independent of age, sex, education, and income (all *p* < 0.05). Physical function scores correlated with self‐reported disability in activities of daily living (*p* < 0.01).	Perceived Severity; Perceived Barriers
Mattei et al. (2010) [10] Allostatic Load Is Associated with Chronic Conditions in the Boston Puerto Rican Health Study	United States Boston, MA (mainland)	Cohort‐based cross‐sectional analysis; Boston Puerto Rican Health Study; logistic regression models for allostatic load and 6 chronic disease outcomes	1,116 Puerto Rican adults aged 45–75 in greater Boston; home‐based interviews and biological samples	Higher allostatic load was associated with greater odds of abdominal obesity (odds ratio 2.1, 95% confidence interval 1.4–3.1), hypertension (odds ratio 3.3, 95% confidence interval 2.2–4.9), diabetes (odds ratio 2.5, 95% confidence interval 1.7–3.6), and self‐reported cardiovascular disease. Associations were comparable to or larger than those for metabolic syndrome across most outcomes.	Perceived Severity; Perceived Susceptibility
Benjamins et al. (2012) [11] Exploring Differences in Self‐Rated Health among Blacks, Whites, Mexicans, and Puerto Ricans	United States Chicago metropolitan area (mainland)	Cross‐sectional; Sinai Improving Community Health Survey; multivariable regression across racial/ethnic groups	Community‐dwelling adults from four racial/ethnic groups (Black, White, Mexican, Puerto Rican) in the Chicago metropolitan area.	Puerto Rican respondents reported lower self‐rated health than Mexican, Black, and White respondents, a pattern that persisted after adjustment for socioeconomic and health‐behavior covariates. Findings supported subgroup‐level disaggregation rather than a pan‐Hispanic or pan‐racial approach to self‐rated health disparities research.	Perceived Susceptibility; Perceived Barriers
Van Rompay et al. (2012) [12] Acculturation and Sociocultural Influences on Dietary Intake and Health Status	United States Boston, MA (mainland)	Cross‐sectional; Boston Puerto Rican Health Study; analysis of covariance and Pearson chi‐square across acculturation quartiles	1,219 Puerto Rican adults aged 45‐75; mean length of mainland stay 34.2 years	Acculturation levels were low despite long mainland residence. Greater English use was associated with higher socioeconomic status, more physical activity, better self‐perceived health, and less central obesity. Poverty status moderated the relationship between acculturation and diet quality.	Perceived Barriers; Self‐Efficacy
Roy, Hughes and Yoshikawa (2013) [13] Intersections Between Nativity, Ethnic Density, and Neighborhood Socioeconomic Status in Puerto Ricans’ Physical Health	United States New York City and Chicago (mainland)	Cross‐sectional multilevel study; Midlife Development in the United States Survey of Minority Groups	449 mainland‐born and island‐born Puerto Rican adults in New York City and Chicago	For island‐born Puerto Ricans, the interaction between ethnic density and neighborhood socioeconomic status was related to self‐rated health, functional limitations, and health symptoms. Island‐born Puerto Ricans in ethnically dense but low socioeconomic status neighborhoods reported the worst health; no comparable interaction was observed for mainland‐born peers.	Perceived Barriers; Perceived Susceptibility
Todorova et al. (2013) [14] Determinants of Self‐Rated Health and the Role of Acculturation	United States Boston, MA (mainland)	Cross‐sectional with moderation analysis; multiple regression; Boston Puerto Rican Health Study	1,357 Puerto Rican adults in the greater Boston area; mean age 57.2 years (SD 7.6)	Number of medical conditions, functional problems, allostatic load, and depressive symptoms were the factors most consistently associated with poor self‐rated health. Acculturation moderated the relationship between health indicators and self‐rated health, and less acculturated individuals in poor health appeared to understate the severity of their condition.	Perceived Susceptibility; Perceived Barriers; Self‐Efficacy
Calo et al. (2014) [15] Factors Associated with Perceived Patient‐Provider Communication Quality among Puerto Ricans	United States National sample (Health Information National Trends Survey)	Cross‐sectional; secondary data; multivariate linear regression	450 Puerto Rican adults from a nationally representative sample	Communication quality was lower among unemployed respondents (*p* = 0.049), those who did not strongly trust providers (*p* = 0.003), and those with higher depressive symptom scores (*p* = 0.036). Higher communication quality was associated with five or more provider visits in the prior year (*p* = 0.023).	Perceived Barriers; Cues to Action
Jimenez et al. (2015) [16] Neighborhood Socioeconomic Context and Change in Allostatic Load Among Older Puerto Ricans	United States Boston, MA (mainland)	Prospective longitudinal cohort; baseline and 2‐year follow‐up; Boston Puerto Rican Health Study; hierarchical linear regression	Approximately 1,000 Puerto Rican adults aged 45‐75 in Boston with longitudinal follow‐up	Higher income relative to neighbors was associated with lower allostatic load at 2‐year follow‐up (beta 0.18, 95% confidence interval 0.05‐0.31, *p* = 0.006). Relative economic disadvantage within a neighborhood, rather than absolute poverty, was the stronger correlate of physiological stress burden over time.	Perceived Barriers; Perceived Susceptibility
Assari and Lankarani (2017) [17] Demographic and Socioeconomic Determinants of Physical and Mental Self‐Rated Health Across 10 Ethnic Groups	United States National sample	Cross‐sectional secondary analysis; Collaborative Psychiatric Epidemiology Surveys 2001–2003; Pearson correlation	18,237 adults from a national household probability sample; Puerto Rican subsample 495 participants	Older age was associated with poorer physical self‐rated health across all groups. For Puerto Ricans, older age was also associated with worse mental self‐rated health. Education and income were associated with better physical and mental self‐rated health across all ethnic groups including Puerto Ricans.	Perceived Barriers; Perceived Susceptibility
Perez and Ailshire (2017) [18] Aging in Puerto Rico: A Comparison of Health Status	Puerto Rico (island) versus United States mainland	Cross‐sectional comparison; 2002 Puerto Rican Elderly Health Conditions Project and Health and Retirement Study	Island Puerto Rican adults aged 60 and older (approximately 4291) versus mainland United States older adults	Island Puerto Ricans were less likely to report poor self‐rated health, heart disease, stroke, and activity‐of‐daily‐living limitations than mainland older adults. However, island Puerto Ricans had higher rates of hypertension and diabetes, and island women had worse outcomes than island men across most measures.	Perceived Severity; Perceived Barriers
Arce‐Ayala et al. (2019) [19] Clinical Profile and Quality of Life of Puerto Ricans with Hereditary Angioedema	Puerto Rico (island)	Cross‐sectional; clinical survey using a Spanish‐adapted quality‐of‐life instrument; comparison with United States population norms	32 Puerto Rican patients with hereditary angioedema	Compared with United States population norms, patients with hereditary angioedema in Puerto Rico reported significantly lower quality of life in both physical (62% of norm) and mental (58% of norm) component scores, with emotional, physical, and social domains most affected.	Perceived Severity; Perceived Susceptibility
Lo et al. (2020) [20] Explaining Chronic Illness and Self‐Rated Health Among Immigrants of Five Hispanic Ethnicities	United States National sample	Cross‐sectional secondary analysis; 17 years of National Health Interview Survey (1999–2015); logistic and linear regression by ethnicity	Hispanic immigrant adults from five ethnic groups including Puerto Ricans; combined sample approximately 1603 across 17 survey years	After adjustment for socioeconomic status, acculturation, and other factors, Puerto Ricans showed higher rates of poor self‐rated health and chronic illness than Cuban and Central and South American groups. The direction and strength of associations between social factors and health outcomes differed meaningfully across Hispanic subgroups.	Perceived Susceptibility; Perceived Barriers
Woo, Wang and Falcon (2020) [21] Unfair Treatment, Coping Strategies, and Depression among Puerto Ricans in Boston	United States Boston, MA (mainland)	Cross‐sectional with moderation analysis; third‐wave Boston Puerto Rican Health Study; logistic regression	963 Puerto Rican adults aged 49–81 in Boston metropolitan area	Perceived unfair treatment was associated with higher odds of depression risk. Four coping strategies, planning, acceptance, humor, and religion, significantly moderated this association, suggesting culturally specific coping serves a protective function against discrimination‐related mental health consequences.	Perceived Susceptibility; Perceived Benefits; Self‐Efficacy
Caraballo‐Cueto and Godreau (2021) [22] Colorism and Health Disparities in Home Countries: The Case of Puerto Rico	Puerto Rico (island)	Cross‐sectional; Puerto Rico Behavioral Risk Factor Surveillance System with an added skin‐color scale; logistic regression and propensity score matching	Puerto Rico Behavioral Risk Factor Surveillance System respondents assessed on a skin‐color scale alongside standard demographic and health items.	Skin color was associated with general health status independent of standard ethno‐racial categories, with darker‐skinned Puerto Ricans reporting worse general health across model specifications, including logistic regression and propensity score matching. Skin color outperformed the standard federal race categories as a predictor of self‐reported health.	Perceived Barriers; Modifying Factors/Structural Inequity
Lee‐Bravatti et al. (2021) [23] Lifestyle Behavioral Factors and Integrative Successful Aging Among Puerto Ricans	United States Boston, MA (mainland)	Prospective cohort; baseline and 2‐year follow‐up; Boston Puerto Rican Health Study; multivariable regression	889 Puerto Rican adults aged 45–75 with data at baseline and 2‐year follow‐up	A lifestyle behavioral score covering nonsmoking, physical activity, diet quality, and adequate sleep was associated with 2‐year improvement in overall successful aging (beta 0.15, 95% confidence interval 0.07–0.24), the physical domain (beta 0.09, 95% confidence interval 0.04–0.13), and the cognitive‐psychosocial domain (beta 0.08, 95% confidence interval 0.02–0.14).	Perceived Benefits; Self‐Efficacy; Perceived Barriers
Buckley, Becker and Burnette (2022) [24] Validation of the Abbreviated Lubben Social Network Scale (LSNS‐6) and Its Association with Self‐Rated Health amongst Older Adults in Puerto Rico	Puerto Rico (island)	Cross‐sectional; face‐to‐face interviews approximately 2 years after Hurricane María; path modeling	154 community‐dwelling older adults (aged 60+) in Puerto Rico, interviewed roughly two years after Hurricane María	The Spanish‐language LSNS‐6 showed acceptable reliability (alpha = 0.74). In path models, the Friends subscale was inversely associated with self‐rated health (beta = −0.31, *p* = 0.045), with a significant indirect effect through psychological sense of community (beta = 0.08, 95% confidence interval 0.01‐0.29); the Family subscale was not significantly associated with self‐rated health.	Perceived Benefits; Modifying Factors/Social Support
Erving and Zajdel (2022) [25] Assessing the Validity of Self‐Rated Health Across Ethnic Groups	United States National sample	Cross‐sectional; National Survey of American Life and National Latino and Asian American Study; ordered logistic regression	8,338 adults across 9 ethnic groups; Puerto Rican subsample approximately 495	Self‐rated health showed within‐group validity for each ethnicity, but cross‐group comparisons were challenged: Puerto Ricans and other minority groups reported worse self‐rated health than non‐Latino Whites even with similar chronic condition burden. Language of interview and years of United States residence partially explained these differences.	Perceived Susceptibility; Perceived Barriers
Santiago‐Pérez et al. (2022) [26] Effect of Chronic Comorbidities on Quality of Life of Gynecologic Cancer Patients in Puerto Rico	Puerto Rico (island)	Cross‐sectional; telephone interviews; multivariate logistic regression	233 women aged 21 and older with a gynecologic cancer diagnosis in Puerto Rico	Most women (90.1%) reported one or more comorbidities in addition to their cancer diagnosis, most commonly cardiovascular disease (63.1%), followed by autoimmune disease (37.3%) and diabetes (33.9%). Comorbidity burden was associated with poorer physical and mental quality‐of‐life items in adjusted models.	Perceived Severity; Perceived Susceptibility; Perceived Barriers
Buckley and Burnette (2023) [27] Psychological Sense of Community, Self‐Rated Health and Quality of Life among Older Adults in Puerto Rico Two Years after Hurricane María	Puerto Rico (island)	Cross‐sectional; face‐to‐face interviews; multiple linear regression using a risk‐and‐resilience framework	154 community‐dwelling older adults (aged 60+) in Puerto Rico, interviewed approximately two years after Hurricane María	Higher psychological sense of community was associated with better quality of life (beta = 0.27, *p* < 0.001), while greater mental health symptoms and poorer self‐rated health were associated with lower quality of life (each *p* < 0.001). Findings support psychological sense of community as a protective factor for older adults following a large‐scale disaster.	Perceived Benefits; Modifying Factors/Social Support
Esteban et al. (2023) [28] Quality of Life and Psychosocial Well‐Being among Intersex‐Identifying Individuals in Puerto Rico: An Exploratory Study	Puerto Rico (island)	Cross‐sectional exploratory pilot; online survey; comparative group design	12 self‐identifying intersex adults and 126 endosex comparison adults in Puerto Rico	This pilot study documented experiences of discrimination and violence among intersex‐identifying adults in Puerto Rico and examined differences in quality of life and psychosocial well‐being relative to an endosex comparison group, providing early descriptive data on a population largely absent from the Puerto Rican health disparities literature.	Perceived Barriers; Perceived Susceptibility
Frontera‐Escudero et al. (2023) [29] Sociodemographic and Health Risk Factors Associated with Health‐Related Quality of Life among Adults Living in Puerto Rico in 2019: A Cross‐Sectional Study	Puerto Rico (island)	Cross‐sectional; 2019 Behavioral Risk Factor Surveillance System, Puerto Rico; prevalence and 95% confidence interval estimation	4944 adults responding to the 2019 Behavioral Risk Factor Surveillance System in Puerto Rico	Poorer health‐related quality of life was concentrated among older adults, those with lower income, and those with chronic conditions, updating population‐level estimates that had not been assessed since 2011 and predate the compounding effects of recent hurricanes, the debt crisis, and the COVID‐19 pandemic.	Perceived Susceptibility; Perceived Severity; Perceived Barriers
Lojo et al. (2023) [30] Ostomy‐Related Quality of Life in Puerto Ricans Living With Inflammatory Bowel Disease: A Prospective Cohort Study	Puerto Rico (island)	Prospective cohort (single quality‐of‐life assessment per patient, recruited 2012–2014); Stoma‐QOL questionnaire; *t*‐test and ANOVA with Tukey post hoc	102 adults living with inflammatory bowel disease and an ostomy in Puerto Rico (59.4% male; 43.1% Crohn's disease; 58.9% ileostomy)	Having an ostomy for more than 40 months was associated with a higher quality‐of‐life score than a shorter duration (59.0 vs. 50.7, *p* = 0.05). Men reported higher quality‐of‐life scores than women (59.94 vs. 50.23, *p* = 0.0019), suggesting a potential target for sex‐specific ostomy education.	Perceived Severity; Perceived Barriers; Self‐Efficacy
McSorley, Tobin and Kuhn (2023) [31] The Relationship Between Political Efficacy and Self‐Rated Health	United States National sample	Cross‐sectional; 2016 Collaborative Multiracial Post‐election Survey; ordered logistic regression	3156 adults: 1486 Mexican, 484 Puerto Rican, 159 Cuban, 1027 non‐Latino White	For Puerto Ricans, lower internal political efficacy was associated with higher self‐rated health, the inverse of the pattern seen in Mexican, Cuban, and non‐Latino White groups. The authors interpreted this as consistent with Puerto Rico's context of sustained political exclusion, which may decouple political engagement from health perception.	Perceived Susceptibility; Perceived Barriers
Reeve et al. (2023) [32] Health‐Related Quality of Life by Race, Ethnicity, and Country of Origin among Cancer Survivors	United States (national, with Puerto Rican country‐of‐origin subgroup)	Population‐based cross‐sectional; PROMIS‐based clustering methodology by race, ethnicity, and country of origin	National sample of adult cancer survivors, disaggregated by self‐reported race, ethnicity, and country of origin including Puerto Rican origin.	Cuban, Dominican, and Puerto Rican cancer survivors were more likely to fall into a ‘very low health‐related quality of life' cluster, roughly 15 points below United States general‐population norms, a disparity obscured when Hispanic survivors are analyzed as a single aggregated category rather than disaggregated by country of origin.	Perceived Severity; Modifying Factors/Race‐Ethnicity‐Country of Origin
Gago et al. (2024) [33] Self‐Rated Health and Medically Diagnosed Chronic Disease Association among Adults in Puerto Rico	Puerto Rico (island)	Cross‐sectional; Puerto Rico Observational Study of Psychosocial, Environmental, and Chronic Disease Trends (PROSPECT); multivariate logistic regression	965 adults aged 30‐75 in the PROSPECT study in Puerto Rico	A single‐item self‐rated health indicator was associated with medically diagnosed chronic disease status after adjustment for socioeconomic, demographic, and behavioral confounders, supporting the validity of self‐rated health as a population‐level chronic disease indicator in this predominantly Latino sample.	Perceived Severity; Perceived Susceptibility
Sheftel and Heiland (2024) [34] The Role of Place of Birth and Residence in Puerto Rican Health Disparities	United States mainland and Puerto Rico island (national survey)	Cross‐sectional secondary analysis; American Community Survey and Puerto Rico Community Survey 2013–2017	More than 2 million Puerto Rican adults aged 40 and older categorized by birthplace and current residence	Mainland‐born and mainland‐residing Puerto Ricans had the lowest disability rates, while island‐born and island‐residing individuals had the highest. Educational attainment explained part of the disparity, and disability prevalence tracked more closely with birthplace than with current residence, consistent with early‐life conditions shaping later‐life health.	Perceived Susceptibility; Perceived Barriers
Kim et al. (2025) [35] COVID‐19 Attitudes and Quality of Life in Older Adults in Puerto Rico: The Mediating Role of Psychological Sense of Community	Puerto Rico (island)	Cross‐sectional; COVID‐19 knowledge, attitudes, and practices survey (2021); mediated linear regression (Hayes PROCESS)	213 Puerto Rican adults aged 60 and older, surveyed in 2021	Both COVID‐19‐related attitudes and psychological sense of community were positively associated with quality of life, with psychological sense of community partially mediating the association between COVID‐19 attitudes and quality of life among older adults during the pandemic.	Perceived Susceptibility; Perceived Benefits; Cues to Action
López‐Vallejo et al. (2025) [36] Examining Health‐Related Quality of Life in Cancer Survivors: Cross‐Sectional Associations with Comorbidities, Navigation Services Use, and Perceived Social Support	Puerto Rico (island)	Cross‐sectional; START‐PR study (2023‐2025); adjusted logistic regression	643 cancer survivors receiving care in Puerto Rico (START‐PR study)	Cancer survivors with at least one comorbidity (most commonly hypertension, diabetes, depression, arthritis, and asthma) were more likely to report poor health‐related quality of life; median quality‐of‐life scores declined with increasing comorbidity count (*p* < 0.001). Patient navigation services and perceived social support did not significantly modify this association.	Perceived Severity; Perceived Benefits; Perceived Barriers
McSorley and Bacong (2025) [37] Health‐Related Quality of Life in the US Territories of Puerto Rico, Guam, and the Virgin Islands	Puerto Rico, Guam, and the Virgin Islands versus the 50 United States	Cross‐sectional secondary analysis; pooled 2021‐2022 Behavioral Risk Factor Surveillance System	Adult respondents to the pooled 2021‐2022 Behavioral Risk Factor Surveillance System from Puerto Rico, Guam, the Virgin Islands, and the 50 states	Residents of the three United States territories, including Puerto Rico, reported worse physical and mental health‐related quality of life than residents of the 50 states collectively, underscoring a structural data gap given how rarely territories are included in national health‐monitoring analyses.	Perceived Susceptibility; Perceived Barriers
Yang, Li and An (2025) [38] Impact of Hurricane Maria on Mental and Physical Health among Puerto Ricans: A Matched Difference‐in‐Differences Analysis	Puerto Rico (island)	Quasi‐experimental repeated cross‐sections; 2016‐2018 Behavioral Risk Factor Surveillance System; propensity score matching with difference‐in‐differences estimation	Puerto Rico Behavioral Risk Factor Surveillance System respondents (2016‐2018), propensity‐matched to comparison respondents to isolate the hurricane's effect	Using a matched difference‐in‐differences design, the study found statistically significant increases of 0.13 and 0.17 self‐reported poor mental and physical health days per month, respectively, following Hurricane Maria, providing quasi‐experimental evidence that a discrete disaster exposure measurably worsened self‐reported health in this population.	Perceived Susceptibility; Perceived Severity; Perceived Barriers; Cues to Action
Vázquez et al. (2026) [39] Health Status of Women Experiencing Homelessness across Diverse Economic and Cultural Contexts: A Comparative Analysis in Nicaragua, Argentina, Puerto Rico, and Spain	Puerto Rico (island), compared with Nicaragua, Argentina, and Spain	Cross‐sectional comparative; structured interviews with women experiencing homelessness across four sites	Women experiencing homelessness recruited in Puerto Rico, compared with parallel samples in Nicaragua, Argentina, and Spain.	Women experiencing homelessness in Puerto Rico reported elevated chronic illness, disability, and poor perceived health relative to population norms, within a broader cross‐national pattern in which economic development level only partially explained differences in health status among homeless women across the four sites.	Perceived Susceptibility; Perceived Severity; Perceived Barriers

### 2.3 Health Belief Model Coding Procedures

The Health Belief Model was used as an organizing framework rather than as a determinant of study eligibility. Studies were coded as reflecting perceived severity when findings concerned chronic disease burden, functional limitation, disability, or physiological stress. Studies were coded as perceived susceptibility when findings concerned vulnerability related to nativity, discrimination, allostatic load, neighborhood disadvantage, or cumulative risk exposure. Studies were coded as perceived barriers when findings concerned poverty, unemployment, low education, language barriers, healthcare access, provider distrust, or neighborhood‐level constraints. Studies were coded as perceived benefits when findings concerned protective behaviors, coping strategies, lifestyle factors, or health‐promoting practices. Studies were coded as self‐efficacy when findings concerned acculturation, language use, coping capacity, or behavioral resources that could influence perceived ability to manage health. Studies were coded as cues to action when findings concerned healthcare visits, provider communication, screening, or interactions with the healthcare system.

When a study addressed more than one construct, multiple Health Belief Model codes were assigned; for example, studies examining both language barriers and provider communication were coded as both perceived barriers and cues to action. Studies were not assigned to a construct unless the reported exposure or interpretation aligned with these predefined decision rules. Ambiguous findings were retained in the descriptive synthesis but were not used to support strong claims about a specific construct unless the interpretation was consistent with the study's own measures and results. A small number of findings did not map cleanly onto the six core constructs, most often where a study's central exposure was a demographic or structural modifier, such as race/ethnicity/country of origin or social support, rather than a health belief per se; these are labeled as modifying factors in Table 3 to preserve transparency rather than force‐fitting them into a construct the original authors did not measure. Coding was performed by the sole author in two iterative passes: an initial pass during data extraction and a second pass after all 33 studies were finalized, to check construct assignments for internal consistency across similar findings. Table 3 documents the construct(s) assigned to each included study and the specific finding that supported each assignment.

**Table 3:** Health belief model coding procedures, decision rules, and basis for construct assignment.

Study (author, year)	Health belief model construct(s) assigned	Basis for assignment (decision rule applied)
Jaen et al. (1998) [7]	Perceived Barriers; Perceived Susceptibility	Barriers coded from distance‐to‐care and language‐access findings; susceptibility coded from age/gender/health‐status associations with dysphoria.
Rao et al. (2007) [8]	Perceived Susceptibility; Perceived Severity; Perceived Barriers	Susceptibility/severity coded from HIV disease‐burden framing; barriers coded from language‐concordance‐of‐care implications.
Castaneda‐Sceppa et al. (2010) [9]	Perceived Severity; Perceived Barriers	Severity coded from chronic‐condition/physical‐function findings; barriers coded from socioeconomic covariates shaping disability risk.
Mattei et al. (2010) [10]	Perceived Severity; Perceived Susceptibility	Severity/susceptibility coded from allostatic‐load‐to‐disease associations reflecting cumulative physiological stress.
Benjamins et al. (2012) [11]	Perceived Susceptibility; Perceived Barriers	Susceptibility coded from subgroup‐level SRH disparity; barriers coded from socioeconomic/access covariates examined alongside ethnicity.
Van Rompay et al. (2012) [12]	Perceived Barriers; Self‐Efficacy	Barriers coded from poverty‐diet interaction; self‐efficacy coded from acculturation‐related behavioral resources (language use, physical activity).
Roy, Hughes and Yoshikawa (2013) [13]	Perceived Barriers; Perceived Susceptibility	Barriers coded from neighborhood‐disadvantage findings; susceptibility coded from nativity‐by‐context vulnerability pattern.
Todorova et al. (2013) [14]	Perceived Susceptibility; Perceived Barriers; Self‐Efficacy	Susceptibility coded from chronic‐condition/allostatic‐load associations; barriers from poverty/education; self‐efficacy from acculturation‐moderation finding.
Calo et al. (2014) [15]	Perceived Barriers; Cues to Action	Barriers coded from employment/trust findings; cues to action coded from provider‐visit‐frequency association.
Jimenez et al. (2015) [16]	Perceived Barriers; Perceived Susceptibility	Barriers/susceptibility coded from relative neighborhood disadvantage predicting rising physiological stress.
Assari and Lankarani (2017) [17]	Perceived Barriers; Perceived Susceptibility	Barriers/susceptibility coded from age and socioeconomic gradient in physical/mental self‐rated health.
Perez and Ailshire (2017) [18]	Perceived Severity; Perceived Barriers	Severity coded from chronic‐disease‐prevalence comparison; barriers coded from island healthcare‐access context.
Arce‐Ayala et al. (2019) [19]	Perceived Severity; Perceived Susceptibility	Severity/susceptibility coded from the disease‐burden‐to‐quality‐of‐life association in a chronic, symptom‐driven condition.
Lo et al. (2020) [20]	Perceived Susceptibility; Perceived Barriers	Susceptibility/barriers coded from subgroup‐specific chronic‐illness and SRH disparities among Hispanic ethnicities.
Woo, Wang and Falcon (2020) [21]	Perceived Susceptibility; Perceived Benefits; Self‐Efficacy	Susceptibility coded from discrimination‐depression association; benefits/self‐efficacy coded from protective coping strategies.
Caraballo‐Cueto and Godreau (2021) [22]	Perceived Barriers; Modifying Factors/Structural Inequity	Barriers coded as a structural‐inequity/discrimination pathway (colorism) shaping self‐reported health independent of standard race categories.
Lee‐Bravatti et al. (2021) [23]	Perceived Benefits; Self‐Efficacy; Perceived Barriers	Benefits/self‐efficacy coded from protective lifestyle‐behavior findings; barriers coded from baseline socioeconomic/behavioral constraints.
Buckley, Becker and Burnette (2022) [24]	Perceived Benefits; Modifying Factors/Social Support	Benefits coded from the protective social‐network‐to‐SRH pathway operating through sense of community.
Erving and Zajdel (2022) [25]	Perceived Susceptibility; Perceived Barriers	Susceptibility/barriers coded from the cross‐group SRH validity gap linked to acculturation‐related measurement factors.
Santiago‐Pérez et al. (2022) [26]	Perceived Severity; Perceived Susceptibility; Perceived Barriers	Severity/susceptibility coded from comorbidity burden; barriers coded from the socioeconomic covariates adjusted for in the quality‐of‐life models.
Buckley and Burnette (2023) [27]	Perceived Benefits; Modifying Factors/Social Support	Benefits coded from the protective sense‐of‐community‐to‐QOL pathway identified after a disaster exposure.
Esteban et al. (2023) [28]	Perceived Barriers; Perceived Susceptibility	Barriers/susceptibility coded from documented discrimination and violence exposure shaping psychosocial well‐being.
Frontera‐Escudero et al. (2023) [29]	Perceived Susceptibility; Perceived Severity; Perceived Barriers	Susceptibility/severity coded from chronic‐condition and age gradients; barriers coded from income‐related disparities in HRQoL.
Lojo et al. (2023) [30]	Perceived Severity; Perceived Barriers; Self‐Efficacy	Severity coded from ostomy‐duration/QOL association; barriers/self‐efficacy coded from the sex‐based education‐gap interpretation offered by the authors.
McSorley, Tobin and Kuhn (2023) [31]	Perceived Susceptibility; Perceived Barriers	Susceptibility/barriers coded from the politically‐exclusion‐linked, subgroup‐specific efficacy‐health pattern.
Reeve et al. (2023) [32]	Perceived Severity; Modifying Factors/Race‐Ethnicity‐Country of Origin	Severity coded from the very‐low‐HRQoL cluster finding; country‐of‐origin modifying factor coded per the study's central disaggregation approach.
Gago et al. (2024) [33]	Perceived Severity; Perceived Susceptibility	Severity/susceptibility coded directly from the self‐rated‐health‐to‐chronic‐disease association central to the study's aim.
Sheftel and Heiland (2024) [34]	Perceived Susceptibility; Perceived Barriers	Susceptibility/barriers coded from birthplace‐linked disability disparities tied to early‐life socioeconomic conditions.
Kim et al. (2025) [35]	Perceived Susceptibility; Perceived Benefits; Cues to Action	Benefits/cues to action coded from the community‐connectedness and health‐attitude pathway to quality of life during a public health emergency.
López‐Vallejo et al. (2025) [36]	Perceived Severity; Perceived Benefits; Perceived Barriers	Severity coded from comorbidity‐burden‐to‐HRQoL gradient; benefits/barriers coded from the (non‐significant) navigation‐services and social‐support moderation tests.
McSorley and Bacong (2025) [37]	Perceived Susceptibility; Perceived Barriers	Susceptibility/barriers coded from the territory‐versus‐state HRQoL gap reflecting structural/jurisdictional disadvantage.
Yang, Li and An (2025) [38]	Perceived Susceptibility; Perceived Severity; Perceived Barriers; Cues to Action	Susceptibility/severity/barriers/cues to action coded from the disaster‐exposure‐to‐health‐decline pathway spanning physical and mental health days.
Vázquez et al. (2026) [39]	Perceived Susceptibility; Perceived Severity; Perceived Barriers	Susceptibility/severity/barriers coded from the elevated chronic‐illness and disability burden documented among an extremely marginalized subgroup.

### 2.4 Quality Appraisal

The author independently evaluated the 33 included studies using the Joanna Briggs Institute critical appraisal tools. Given the variation in study designs, the Checklist for Analytical Cross‐Sectional Studies was applied to 29 records, including one quasi‐experimental study that applied a matched difference‐in‐differences design to repeated cross‐sectional surveillance data; the Checklist for Cohort Studies was applied to 4 longitudinal or cohort‐derived records. Evaluation criteria focused on sampling representativeness, measurement validity of self‐reported health, and statistical management of confounding variables. The author performed two iterative rounds of appraisal to improve internal consistency. Appraisal was based on the published record for each study; where a checklist item could not be confidently determined from the available text, it was conservatively rated Unclear rather than assumed to be met, consistent with Joanna Briggs Institute guidance for insufficient information. Detailed item‐level appraisal results for the analytical cross‐sectional and quasi‐experimental studies are presented in Table 4. Detailed item‐level appraisal results for the cohort studies are presented in Table 5.

**Table 4:** Joanna Briggs Institute Critical Appraisal—analytical cross‐sectional and quasi‐experimental studies (*n* = 29).

Study (author, year)	N/Sample	CS1 inclusion criteria	CS2 setting description	CS3 exposure measurement	CS4 outcome criteria	CS5 confounding	CS6 outcome validity	CS7 statistics	CS8 response rate	Overall quality	Critical appraisal notes
Jaen et al. (1998) [7]	*N* = 704	Yes	Yes	Yes	Yes	Yes	Yes	Yes	Unclear	Moderate Quality	Validated dysphoria scale; community‐based sample. Response rate not reported; barrier items locally developed without formal validation.
Rao et al. (2007) [8]	*N* not separately reported for PR subsample	Yes	Yes	Unclear	Yes	Unclear	Yes	Yes	Unclear	Moderate Quality	Validated HIV‐specific HRQoL instrument administered by language; cross‐site comparison. Confounder control and response rate not detailed in the available record; Puerto Rico subsample size not separately reported.
Castaneda‐Sceppa et al. (2010) [9]	*N* = 1357	Yes	Yes	Yes	Yes	Yes	Yes	Yes	Yes	High Quality	Large community cohort; validated performance measures; regression adjusted for age, sex, education, income, and lifestyle. Cross‐sectional design limits causal inference.
Benjamins et al. (2012) [11]	*N* not separately reported (four‐group community survey)	Yes	Yes	Yes	Yes	Yes	Unclear	Yes	Unclear	Moderate Quality	Locally representative community health survey with multi‐group comparison. Response rate and single‐item outcome validity across subgroups not fully detailed in the available record.
Van Rompay et al. (2012) [12]	*N* = 1219	Yes	Yes	Yes	Yes	Yes	Yes	Yes	Yes	High Quality	Culturally tailored food frequency questionnaire; poverty interaction tested. Acculturation measured by proxy (language use); cross‐sectional design.
Roy, Hughes and Yoshikawa (2013) [13]	*N* = 449	Yes	Yes	Yes	Yes	Yes	Yes	Yes	Unclear	Moderate Quality	Multilevel analysis; nativity by neighborhood interaction is a key strength. Small sample limits power for complex interaction terms; response rate not reported.
Todorova et al. (2013) [14]	*N* = 1357	Yes	Yes	Yes	Yes	Yes	Yes	Yes	Yes	High Quality	Moderation analysis with biomarker allostatic load included. Cross‐sectional design; self‐rated health may mask disparities in less acculturated individuals.
Calo et al. (2014) [15]	*N* = 450	Yes	Yes	Unclear	Yes	Yes	Yes	Yes	Unclear	Moderate Quality	Composite communication scale (alpha = 0.87); small Puerto Rican subsample from a national survey. Health Information National Trends Survey response rate not reported; limited confounder control.
Assari and Lankarani (2017) [17]	*N* = 18,237 (PR = 495)	Yes	Yes	Yes	Yes	Yes	Unclear	Yes	Yes	Moderate‐High Quality	Physical and mental self‐rated health distinguished; 10 ethnic groups compared. Puerto Rican subsample N = 495; single‐item outcome; Pearson correlation is exploratory only.
Perez and Ailshire (2017) [18]	*N* ~ 4291 (PR) versus HRS	Yes	Yes	Unclear	Yes	Yes	Yes	Yes	Unclear	Moderate Quality	Island versus mainland comparison; gender‐stratified analysis. Two different survey instruments introduce comparability concerns; exposure not identically measured across groups.
Arce‐Ayala et al. (2019) [19]	*N* = 32	Yes	Yes	Yes	Yes	Unclear	Yes	Yes	Unclear	Moderate Quality	Small, well‐characterized disease‐specific clinical sample with a validated quality‐of‐life instrument. Small sample size limits precision; confounder adjustment and response rate not detailed.
Lo et al. (2020) [20]	*N* ~ 1603 (pooled)	Yes	Yes	Yes	Yes	Yes	Yes	Yes	Yes	High Quality	Seventeen‐year pooled National Health Interview Survey data; ethnicity‐stratified regression. Secular trends across 17 years may confound results.
Woo, Wang and Falcon (2020) [21]	*N *= 963	Yes	Yes	Yes	Yes	Yes	Yes	Yes	Yes	High Quality	Twelve coping strategies systematically tested; validated instruments. Cross‐sectional wave; attrition from Boston Puerto Rican Health Study baseline not addressed.
Caraballo‐Cueto and Godreau (2021) [22]	*N* (PR BRFSS, skin‐color subsample) not separately reported	Yes	Yes	Yes	Yes	Yes	Unclear	Yes	Yes	Moderate‐High Quality	Population‐based surveillance sample with an innovative skin‐color measure and dual analytic approach (regression and matching). Validity of the newly added skin‐color item was not independently psychometrically tested.
Buckley, Becker and Burnette (2022) [24]	*N* = 154	Yes	Yes	Yes	Yes	Unclear	Yes	Yes	Unclear	Moderate Quality	Culturally adapted, psychometrically tested social‐network measure with a clear post‐disaster rationale. Non‐probability sample; broader confounder adjustment beyond the path model not detailed.
Erving and Zajdel (2022) [25]	*N* = 8338 (PR ~ 495)	Yes	Yes	Yes	Yes	Yes	Unclear	Yes	Yes	Moderate‐High Quality	Cross‐ethnic validity framework; ordered logistic regression. Puerto Rican subsample (N approximately 495) limits power; acculturation proxy partially unclear.
Santiago‐Pérez et al. (2022) [26]	*N* = 233	Yes	Yes	Yes	Yes	Yes	Yes	Yes	Unclear	Moderate Quality	Clinically well‐defined disease population with structured telephone interviews and adjusted regression. Response rate for telephone recruitment not detailed in the available record.
Buckley and Burnette (2023) [27]	*N* = 154	Yes	Yes	Yes	Yes	Yes	Yes	Yes	Unclear	Moderate Quality	Risk‐and‐resilience framework with validated community and quality‐of‐life measures. Non‐probability sample; drawn from the same post‐María cohort as the companion LSNS‐6 study, so findings are not fully independent.
Esteban et al. (2023) [28]	*N* = 12 intersex; *N* = 126 endosex comparison	Yes	Yes	Unclear	Yes	Unclear	Unclear	Yes	Unclear	Moderate Quality	First empirical Puerto Rico‐based study of this population; clearly described exploratory pilot design. Very small intersex subgroup (*n* = 12) limits statistical power and precision; confounding not formally addressed given pilot scope.
Frontera‐Escudero et al. (2023) [29]	*N* = 4944	Yes	Yes	Yes	Yes	Yes	Yes	Yes	Yes	High Quality	Large, population‐representative Behavioral Risk Factor Surveillance System sample with standard validated items. Cross‐sectional design limits causal inference regarding chronic‐condition and income pathways.
McSorley, Tobin and Kuhn (2023) [31]	*N* = 484 (PR subsample)	Yes	Yes	Yes	Yes	Yes	Yes	Yes	Unclear	Moderate Quality	First empirical study of political efficacy and self‐rated health in Puerto Rican adults. Puerto Rican subsample N = 484; parent survey response rate not reported; cross‐sectional.
Reeve et al. (2023) [32]	*N* not separately reported for PR‐origin subgroup	Yes	Yes	Yes	Yes	Yes	Yes	Yes	Unclear	Moderate‐High Quality	Methodologically innovative disaggregation by country of origin within a large national survivorship sample. Puerto Rican‐origin subsample size and response rate not separately detailed in the available record.
Gago et al. (2024) [33]	*N* = 965	Yes	Yes	Yes	Yes	Yes	Yes	Yes	Unclear	High Quality	Purpose‐built observational cohort with adjusted multivariate models directly testing self‐rated health validity. Response rate for the parent PROSPECT recruitment not detailed in the available record.
Sheftel and Heiland (2024) [34]	*N* > 2 million	Yes	Yes	Yes	Yes	Yes	Yes	Yes	Yes	High Quality	Very large nationally representative sample; nativity‐by‐residence design is an innovative structural approach. Cross‐sectional; disability measured via American Community Survey items only.
Kim et al. (2025) [35]	*N* = 213	Yes	Yes	Yes	Yes	Unclear	Yes	Yes	Unclear	Moderate Quality	Purpose‐built pandemic‐era survey of older Puerto Rican adults with formal mediation modeling. Non‐probability sample; response rate and full confounder set not detailed in the available record.
López‐Vallejo et al. (2025) [36]	*N* = 643	Yes	Yes	Yes	Yes	Yes	Yes	Yes	Unclear	High Quality	Large, purpose‐built cancer survivorship cohort with a validated HRQoL instrument and fully adjusted models. Response rate for START‐PR recruitment not detailed in the available record.
McSorley and Bacong (2025) [37]	*N* (territories, pooled) not separately reported for PR	Yes	Yes	Yes	Yes	Yes	Yes	Yes	Unclear	High Quality	Large, population‐representative, multi‐territory Behavioral Risk Factor Surveillance System sample following STROBE reporting guidance. Puerto Rico‐specific effect estimates (*vs.* the pooled 3‐territory estimate) not separately detailed in the available record.
Yang, Li and An (2025) [38]	*N* (BRFSS 2016–2018, matched)	Yes	Yes	Yes	Yes	Yes	Yes	Yes	Unclear	Moderate‐High Quality	Quasi‐experimental matched difference‐in‐differences design strengthens causal interpretation relative to purely cross‐sectional studies in this review, though it is based on repeated independent cross‐sections rather than a followed cohort. Response rate for underlying Behavioral Risk Factor Surveillance System waves not detailed in the available record.
Vázquez et al. (2026) [39]	N (PR site) not separately reported	Yes	Yes	Unclear	Yes	Unclear	Unclear	Yes	Unclear	Moderate Quality	Structured comparative design extending a well‐established cross‐national homelessness research program to a Puerto Rico site for the first time. Puerto Rico‐specific sample size, response rate, and confounder adjustment not separately detailed in the available record.

*Note:* Response codes: Yes = criterion met; No = criterion not met; Unclear = insufficient information to judge; NA = not applicable to this study design. Ratings were assigned conservatively (Unclear where the published record did not permit confident judgment), consistent with Joanna Briggs Institute guidance (Section 2.4).

**Table 5:** Joanna Briggs Institute Critical Appraisal—cohort and cohort‐derived studies (*n* = 4).

Study (author, year)	*N*/sample	CO1 group similarity	CO2 exposure assignment	CO3 exposure validity	CO4 confounding	CO5 outcome validity	CO6 follow‐up duration	CO7 follow‐up complete	CO8 withdrawal outcomes	CO9 outcome measurement	CO10 statistics	CO11 selective reporting	Overall quality	Critical appraisal notes
Mattei et al. (2010) [10]	*N* = 1116	NA	NA	Yes	Yes	Yes	NA	NA	NA	Yes	Yes	Yes	Moderate‐High Quality	Cross‐sectional analysis within cohort baseline; follow‐up items not applicable. Ten‐biomarker allostatic load is a key strength; logistic regression with disease‐specific models is appropriate.
Jimenez et al. (2015) [16]	*N* ~ 1000	Yes	Yes	Yes	Yes	Yes	Yes	Yes	Yes	Yes	Yes	Yes	High Quality	Hierarchical linear regression; relative income as a novel predictor of allostatic load change. Attrition details limited; neighborhood‐level Census data linked appropriately.
Lee‐Bravatti et al. (2021) [23]	*N* = 889	Yes	Yes	Yes	Yes	Yes	Yes	Yes	Yes	Yes	Yes	Yes	High Quality	Prospective 2‐year follow‐up; composite lifestyle score from validated measures. Attrition from baseline (approximately 41%); attrition analysis not fully reported.
Lojo et al. (2023) [30]	*N* = 102	Yes	Yes	Yes	Yes	Unclear	Yes	Yes	NA	NA	NA	Yes	Moderate Quality	Disease‐specific cohort recruited from a single specialty center with a validated ostomy quality‐of‐life instrument. Quality of life was assessed at a single time point per patient rather than with repeated within‐subject follow‐up, so several cohort‐specific follow‐up items are not applicable; broader confounder adjustment beyond duration and sex was not detailed.

*Note:* Response codes: Yes = criterion met; No = criterion not met; Unclear = insufficient information to judge; NA = not applicable to this study design. Follow‐up‐specific items (CO6‐CO8) are marked NA for studies in which the outcome was assessed at a single time point per participant rather than through repeated within‐subject follow‐up.

## 3 Results

### 3.1 Study Selection

The database search returned 2,059 records across nine databases (Table 1). A three‐stage deduplication process removed 788 duplicate records (704 by DOI match, 82 by title match, and 2 by abstract match), leaving 1271 unique records for title and abstract screening. Of those, 1233 were excluded at screening for the following reasons: out of scope or irrelevant outcome (*n* = 497), ineligible study design or publication type (*n* = 326), ineligible population or no Puerto Rican‐specific data (*n* = 245), and condition‐specific clinical or treatment outcomes outside the review's scope (*n* = 165). The remaining 38 reports were assessed for full‐text eligibility; 33 met inclusion criteria and were retained for qualitative synthesis after five exclusions at the full‐text stage (Section 2.2). The complete selection process is depicted in Figure 1.

### 3.2 Study Characteristics

Twenty‐nine of the 33 included studies used cross‐sectional or quasi‐experimental repeated‐cross‐section designs. Four used cohort or cohort‐derived designs: three drawn from the Boston Puerto Rican Health Study and one from a Puerto Rico‐based inflammatory bowel disease registry. Geographic settings included Boston (Massachusetts), New York City, Chicago, and Puerto Rico, as well as nationally representative United States samples and, for one comparative study of women experiencing homelessness, sites in Nicaragua, Argentina, and Spain alongside Puerto Rico. Sample sizes ranged from 12 participants in a pilot study of intersex‐identifying adults to more than 2 million in a national administrative‐survey analysis. The Population, Exposure, and Outcome framework summary for all included studies is presented in Table 6.

**Table 6:** Population, exposure, and outcome (PEO) framework summary.

Author(s) and year	Population (P)	Exposure (E)	Outcome (O)
Jaen et al. (1998) [7]	704 adults living in a predominantly Puerto Rican urban community on the United States mainland.	Barriers to healthcare including distance to providers, language differences, and lack of health insurance, along with age and gender.	Self‐reported health status and dysphoria measured as key outcomes, with health status associated with psychological wellbeing in this community.
Rao et al. (2007) [8]	Adults living with HIV/AIDS in the continental United States and Puerto Rico, surveyed in English or Spanish.	Language of survey administration, geographic site (mainland vs. Puerto Rico), and HIV disease‐related symptom burden.	Health‐related quality of life, measured with a validated HIV‐specific instrument administered in the participant's preferred language.
Castaneda‐Sceppa et al. (2010) [9]	1357 Puerto Rican adults aged 45–75 living in Boston, Massachusetts. A community‐based sample drawn specifically from the Puerto Rican population.	Chronic conditions including diabetes, heart disease, arthritis, and depression, along with obesity, age, sex, income, and education level.	Self‐reported disability in daily activities and overall physical health status, measured through performance‐based and self‐report tools.
Mattei et al. (2010) [10]	1116 Puerto Rican adults aged 45–75 living in the greater Boston area who completed home‐based interviews and provided biological samples.	Allostatic load, a composite physiological score reflecting cumulative stress from chronic social disadvantage, poverty, and discrimination.	Self‐reported chronic conditions including cardiovascular disease and arthritis, along with chronic disease burden as self‐reported health outcomes.
Benjamins et al. (2012) [11]	Community‐dwelling Black, White, Mexican, and Puerto Rican adults sampled through a local community health survey in the Chicago metropolitan area.	Racial/ethnic subgroup membership together with socioeconomic status, health behaviors, and access‐to‐care indicators.	Single‐item self‐rated health, compared across the four racial/ethnic subgroups.
Van Rompay et al. (2012) [12]	1219 Puerto Rican adults aged 45–75 living in Boston, Massachusetts, with an average of 34 years of mainland residence.	Acculturation level, English language use, poverty status, dietary quality, physical activity, and alcohol use.	Self‐perceived health status reported as an outcome alongside dietary and behavioral health indicators.
Roy, Hughes and Yoshikawa (2013) [13]	449 mainland‐born and island‐born Puerto Rican adults living in New York City and Chicago.	Neighborhood ethnic density, neighborhood socioeconomic status, and nativity as structural and contextual factors shaping health.	Self‐reported physical health status, functional limitations, and health symptoms measured directly as primary outcomes.
Todorova et al. (2013) [14]	1357 Puerto Rican adults with a mean age of 57 years living in Boston, Massachusetts.	Acculturation, number of medical conditions, allostatic load, depressive symptoms, poverty, education, social support, and lifestyle behaviors.	Self‐rated health measured as the primary outcome using multiple regression analysis.
Calo et al. (2014) [15]	450 Puerto Rican adults drawn from a nationally representative Health Information National Trends Survey sample.	Employment status, trust in healthcare providers, depression severity, and frequency of healthcare visits.	Perceived quality of patient‐provider communication, which directly shapes how Puerto Ricans engage with the healthcare system and report their health.
Jimenez et al. (2015) [16]	Approximately 1000 Puerto Rican adults aged 45–75 in Boston, Massachusetts with two‐year longitudinal follow‐up data.	Neighborhood socioeconomic status, an individual's income relative to their neighbors, length of residence, and individual‐level socioeconomic position.	Change in allostatic load over time as a physiological measure of cumulative health burden, linked to self‐reported health status in this population.
Assari and Lankarani (2017) [17]	18,237 adults from a national probability sample including 495 Puerto Rican participants analyzed as a distinct ethnic subgroup.	Age, sex, education, income, and body mass index as demographic and socioeconomic determinants of health.	Physical self‐rated health and mental self‐rated health measured separately as primary outcomes across all ethnic groups including Puerto Ricans.
Perez and Ailshire (2017) [18]	Island Puerto Rican adults aged 60 and older from the Puerto Rican Elderly Health Conditions Project, compared to mainland US older adults.	Geographic residence on the island versus the mainland, gender, chronic conditions including hypertension and diabetes, and healthcare access.	Self‐rated health, activity of daily living limitations, and multiple self‐reported health status measures.
Arce‐Ayala et al. (2019) [19]	32 Puerto Rican patients with a confirmed diagnosis of hereditary angioedema.	Disease‐specific symptom burden, attack frequency, and treatment access for a rare hereditary condition.	Disease‐specific and generic health‐related quality of life, benchmarked against United States population norms.
Lo et al. (2020) [20]	Puerto Rican adults drawn from 17 years of the National Health Interview Survey, analyzed as a distinct ethnic subgroup.	Acculturation, social support, income, education, lifestyle behaviors, and Hispanic ethnicity as a moderating structural factor.	Self‐rated health and chronic illness burden measured as primary outcomes and analyzed separately for the Puerto Rican subgroup.
Woo, Wang and Falcon (2020) [21]	963 Puerto Rican adults aged 49‐81 living in the Boston, Massachusetts area.	Perceived unfair treatment and discrimination, along with 12 types of coping strategies including religion, humor, planning, and acceptance.	Self‐reported depression risk and mental health status measured using a validated clinical scale.
Caraballo‐Cueto and Godreau (2021) [22]	Adult respondents to the Puerto Rico Behavioral Risk Factor Surveillance System with an added skin‐color scale.	Self‐reported skin color/colorism, alongside standard sociodemographic covariates including age, sex, and income.	General self‐reported health status, compared across skin‐color categories using logistic regression and propensity score matching.
Lee‐Bravatti et al. (2021) [23]	889 Puerto Rican adults aged 45–75 from the Boston Puerto Rican Health Study with two‐year follow‐up data.	A comprehensive lifestyle behavioral score covering physical activity, diet quality, sleep adequacy, smoking status, and sedentary behavior.	Integrative successful aging score across physical and cognitive‐psychosocial health domains, reflecting overall health status over time.
Buckley, Becker and Burnette (2022) [24]	154 community‐dwelling older adults (aged 60 and older) in Puerto Rico approximately 2 years after Hurricane María.	Social network size and contact frequency with friends and family (LSNS‐6), and psychological sense of community.	Self‐rated health and its statistical pathway through social isolation and community connectedness.
Erving and Zajdel (2022) [25]	A national household sample of 8338 adults including approximately 495 Puerto Rican participants analyzed as a distinct subgroup.	Ethnicity, acculturation level, language of interview, and years of United States residence as factors shaping how health is perceived and reported.	Self‐rated health measured through ordered logistic regression, with validity of the measure examined within and across ethnic groups.
Santiago‐Pérez et al. (2022) [26]	233 women aged 21 and older with a gynecologic cancer diagnosis in Puerto Rico.	Chronic comorbidities including cardiovascular disease, autoimmune disease, and diabetes, alongside sociodemographic covariates.	Quality of life measured across six physical and mental health items, analyzed via adjusted prevalence odds ratios.
Buckley and Burnette (2023) [27]	154 community‐dwelling older adults (aged 60 and older) in Puerto Rico approximately 2 years after Hurricane María (same cohort as the related LSNS‐6 validation study).	Psychological sense of community, self‐rated health, and mental health symptoms as predictors of overall well‐being.	Quality of life (EUROHIS‐QOL 8‐item index), examined as the primary outcome using multiple linear regression.
Esteban et al. (2023) [28]	12 self‐identifying intersex adults and 126 endosex comparison adults recruited through an online survey in Puerto Rico.	Intersex identity status, alongside self‐reported experiences of discrimination and violence.	Quality of life, psychological well‐being, and social well‐being, compared between intersex‐identifying and endosex participants.
Frontera‐Escudero et al. (2023) [29]	4944 adults living in Puerto Rico who responded to the 2019 Behavioral Risk Factor Surveillance System.	Sociodemographic characteristics (age, income, education) and health risk factors (chronic conditions, health behaviors).	Health‐related quality of life, estimated as prevalence with 95% confidence intervals across sociodemographic and health‐risk strata.
Lojo et al. (2023) [30]	102 adults living with inflammatory bowel disease and an ostomy, recruited from a specialty IBD center in San Juan, Puerto Rico.	Duration of time living with the ostomy, sex, IBD diagnosis type, and ostomy type.	Ostomy‐related quality of life, measured with the Stoma‐QOL questionnaire.
McSorley, Tobin and Kuhn (2023) [31]	484 Puerto Rican adults from a nationally representative post‐election survey analyzed as a distinct subgroup.	Internal and external political efficacy as a structural and psychosocial factor shaping how individuals perceive their own health.	Self‐rated health measured as the primary dependent variable using ordered logistic regression.
Reeve et al. (2023) [32]	National sample of adult cancer survivors, including a Puerto Rican country‐of‐origin subgroup analyzed apart from other Hispanic‐origin groups.	Race, ethnicity, and self‐reported country of origin as an innovative disaggregation approach beyond standard Hispanic/non‐Hispanic coding.	Health‐related quality of life clusters, benchmarked against United States general‐population norms.
Gago et al. (2024) [33]	965 adults aged 30–75 years participating in the PROSPECT study in Puerto Rico.	Medically diagnosed chronic disease status (ever *vs.* never), adjusted for socioeconomic, demographic, and behavioral confounders.	Single‐item self‐rated health (excellent/very good, good, or fair/poor), examined for its association with chronic disease status.
Sheftel and Heiland (2024) [34]	More than 2 million Puerto Rican adults aged 40 and older from the American Community Survey and Puerto Rico Community Survey.	Birthplace on the island versus the mainland, current place of residence, educational attainment, and early‐life socioeconomic disadvantage.	Self‐reported disability prevalence as the primary outcome, compared across four birthplace and residence combinations.
Kim et al. (2025) [35]	213 Puerto Rican adults aged 60 and older, surveyed on COVID‐19 knowledge, attitudes, and practices in 2021.	COVID‐19‐related attitudes and psychological sense of community, modeled as predictor and mediator respectively.	Quality of life, examined through a mediated linear regression model.
López‐Vallejo et al. (2025) [36]	643 cancer survivors receiving care in Puerto Rico, enrolled in the START‐PR study.	Chronic comorbidity burden, patient navigation services use, and perceived social support.	Health‐related quality of life (FACT‐G), adjusted for demographic, clinical, and behavioral covariates.
McSorley and Bacong (2025) [37]	Adult respondents to the pooled 2021–2022 Behavioral Risk Factor Surveillance System, comparing Puerto Rico, Guam, and the Virgin Islands with the 50 states.	Territory versus state residency status as a structural/jurisdictional exposure.	Physical and mental health‐related quality of life, compared between territories and the 50 states.
Yang, Li and An (2025) [38]	Adult respondents to the Puerto Rico Behavioral Risk Factor Surveillance System surveyed before and after Hurricane Maria (2016‐2018), propensity‐matched to a comparison group.	Hurricane Maria exposure (September 2017) as a discrete environmental/disaster event, analyzed with a difference‐in‐differences design.	Self‐reported days of poor mental and physical health per month, before versus after the hurricane.
Vázquez et al. (2026) [39]	Women experiencing homelessness in Puerto Rico, compared with parallel samples in Nicaragua, Argentina, and Spain.	Homelessness status and site/country context (differing economic development and social‐welfare structures).	Self‐reported health status, chronic illness, and disability, compared across the four national/territorial contexts.

### 3.3 Risk of Bias Across Studies

Quality appraisal using the Joanna Briggs Institute critical appraisal tools indicated a range of methodological quality across the 33 included studies. Twelve studies were rated High Quality, six \were rated Moderate‐High Quality, and fifteen were rated Moderate Quality. No study was excluded on the basis of quality rating alone; all ratings are reported alongside findings to support transparency.

Studies rated High Quality were disproportionately those with large probability‐based or purpose‐built samples, validated outcome measures, and documented multivariable confounder adjustment (Table 7). The most common sources of a lower rating were an unreported survey response rate and, in secondary‐data analyses of national surveys, a limited ability to verify confounder control beyond what was described in the published record; for the smallest and most exploratory studies, including the pilot study of intersex‐identifying adults (Section 4.10), limited statistical power was an additional consideration. Detailed item‐level appraisal results are presented in Tables 4 and 5.

**Table 7:** Quality appraisal summary—All 33 included studies.

Study	Study design	Tool applied	Overall quality	Key strength	Key limitation
Jaen et al. (1998) [7]	Cross‐sectional community survey	Cross‐Sectional Checklist	Moderate Quality	Validated dysphoria scale; community‐based sample.	Response rate not reported; barrier items locally developed without formal validation.
Rao et al. (2007) [8]	Cross‐sectional	Cross‐Sectional Checklist	Moderate Quality	Validated HIV‐specific HRQoL instrument administered by language; cross‐site mainland/island comparison.	Confounder control and response rate not detailed; Puerto Rico subsample size not separately reported.
Castaneda‐Sceppa et al. (2010) [9]	Cross‐sectional	Cross‐Sectional Checklist	High Quality	Large community cohort; validated performance measures; regression adjusted for age, sex, education, income, and lifestyle.	Cross‐sectional design limits causal inference.
Mattei et al. (2010) [10]	Cohort‐based cross‐sectional analysis	Cohort Checklist	Moderate‐High Quality	Ten‐biomarker allostatic load is a key strength; logistic regression with disease‐specific models appropriate.	Cross‐sectional analysis within cohort baseline; follow‐up items not applicable; causal direction unestablished.
Benjamins et al. (2012) [11]	Cross‐sectional	Cross‐Sectional Checklist	Moderate Quality	Locally representative multi‐group community health survey enabling direct subgroup comparison.	Response rate and single‐item outcome validity across subgroups not fully detailed.
Van Rompay et al. (2012) [12]	Cross‐sectional	Cross‐Sectional Checklist	High Quality	Culturally tailored food frequency questionnaire; poverty interaction tested.	Acculturation measured by proxy (language use); cross‐sectional design.
Roy, Hughes and Yoshikawa (2013) [13]	Cross‐sectional multilevel study	Cross‐Sectional Checklist	Moderate Quality	Multilevel analysis; nativity by neighborhood interaction is a key strength.	Small sample limits power for complex interaction terms; response rate not reported.
Todorova et al. (2013) [14]	Cross‐sectional with moderation analysis	Cross‐Sectional Checklist	High Quality	Moderation analysis with biomarker allostatic load included.	Cross‐sectional; self‐rated health may mask disparities in less acculturated individuals.
Calo et al. (2014) [15]	Cross‐sectional	Cross‐Sectional Checklist	Moderate Quality	Composite communication scale (alpha = 0.87); national survey sampling frame.	Small Puerto Rican subsample; Health Information National Trends Survey response rate not reported; limited confounder control.
Jimenez et al. (2015) [16]	Prospective longitudinal cohort	Cohort Checklist	High Quality	Hierarchical linear regression; relative income as a novel predictor of allostatic load change.	Attrition details limited; neighborhood effect not independently significant.
Assari and Lankarani (2017) [17]	Cross‐sectional secondary analysis	Cross‐Sectional Checklist	Moderate‐High Quality	Physical and mental self‐rated health distinguished; 10 ethnic groups compared.	Puerto Rican subsample N = 495; single‐item outcome; Pearson correlation is exploratory only.
Perez and Ailshire (2017) [18]	Cross‐sectional comparison	Cross‐Sectional Checklist	Moderate Quality	Island versus mainland comparison; gender‐stratified analysis.	Two different survey instruments; exposure not identically measured across groups.
Arce‐Ayala et al. (2019) [19]	Cross‐sectional	Cross‐Sectional Checklist	Moderate Quality	Disease‐specific clinical sample with a validated quality‐of‐life instrument benchmarked to US norms.	Small sample size limits precision; confounder adjustment and response rate not detailed.
Lo et al. (2020) [20]	Cross‐sectional secondary analysis	Cross‐Sectional Checklist	High Quality	Seventeen‐year pooled National Health Interview Survey data; ethnicity‐stratified regression.	Secular trends across 17 years may confound results.
Woo, Wang and Falcon (2020) [21]	Cross‐sectional with moderation analysis	Cross‐Sectional Checklist	High Quality	Twelve coping strategies systematically tested; validated instruments.	Cross‐sectional wave; attrition from Boston Puerto Rican Health Study baseline not addressed.
Caraballo‐Cueto and Godreau (2021) [22]	Cross‐sectional	Cross‐Sectional Checklist	Moderate‐High Quality	Population‐based surveillance sample with an innovative skin‐color measure and dual analytic approach (regression and matching).	Validity of the newly added skin‐color item was not independently psychometrically tested.
Lee‐Bravatti et al. (2021) [23]	Prospective cohort	Cohort Checklist	High Quality	Prospective 2‐year follow‐up; composite lifestyle score from validated measures.	Attrition from baseline (approximately 41%); attrition analysis not fully reported.
Buckley, Becker and Burnette (2022) [24]	Cross‐sectional	Cross‐Sectional Checklist	Moderate Quality	Culturally adapted, psychometrically tested social‐network measure with a clear post‐disaster rationale.	Non‐probability sample; broader confounder adjustment beyond the path model not detailed.
Erving and Zajdel (2022) [25]	Cross‐sectional	Cross‐Sectional Checklist	Moderate‐High Quality	Cross‐ethnic validity framework; ordered logistic regression.	Puerto Rican subsample (N ~ 495) limits power; acculturation proxy partially unclear.
Santiago‐Pérez et al. (2022) [26]	Cross‐sectional	Cross‐Sectional Checklist	Moderate Quality	Clinically well‐defined disease population with structured interviews and adjusted regression.	Response rate for telephone recruitment not detailed.
Buckley and Burnette (2023) [27]	Cross‐sectional	Cross‐Sectional Checklist	Moderate Quality	Risk‐and‐resilience framework with validated community and quality‐of‐life measures.	Non‐probability sample drawn from the same post‐Maria cohort as the companion LSNS‐6 study.
Esteban et al. (2023) [28]	Cross‐sectional exploratory pilot	Cross‐Sectional Checklist	Moderate Quality	First empirical Puerto Rico‐based study of this population; clearly described exploratory pilot design.	Very small intersex subgroup (*n* = 12) limits statistical power and precision.
Frontera‐Escudero et al. (2023) [29]	Cross‐sectional	Cross‐Sectional Checklist	High Quality	Large, population‐representative Behavioral Risk Factor Surveillance System sample with standard validated items.	Cross‐sectional design limits causal inference regarding chronic‐condition and income pathways.
Lojo et al. (2023) [30]	Prospective cohort (single quality‐of‐life assessment per patient, recruited 2012‐2014)	Cohort Checklist	Moderate Quality	Disease‐specific cohort from a single specialty center with a validated ostomy quality‐of‐life instrument.	Quality of life assessed at a single time point per patient; several follow‐up items not applicable.
McSorley, Tobin and Kuhn (2023) [31]	Cross‐sectional	Cross‐Sectional Checklist	Moderate Quality	First empirical study of political efficacy and self‐rated health in Puerto Rican adults.	Puerto Rican subsample *N* = 484; parent survey response rate not reported; cross‐sectional.
Reeve et al. (2023) [32]	Population‐based cross‐sectional	Cross‐Sectional Checklist	Moderate‐High Quality	Methodologically innovative disaggregation by country of origin within a large national survivorship sample.	Puerto Rican‐origin subsample size and response rate not separately detailed.
Gago et al. (2024) [33]	Cross‐sectional	Cross‐Sectional Checklist	High Quality	Purpose‐built observational cohort with adjusted multivariate models directly testing self‐rated health validity.	Response rate for the parent PROSPECT recruitment not detailed.
Sheftel and Heiland (2024) [34]	Cross‐sectional secondary analysis	Cross‐Sectional Checklist	High Quality	Very large nationally representative sample; nativity‐by‐residence design is an innovative structural approach.	Cross‐sectional; disability measured via American Community Survey items only.
Kim et al. (2025) [35]	Cross‐sectional	Cross‐Sectional Checklist	Moderate Quality	Purpose‐built pandemic‐era survey of older Puerto Rican adults with formal mediation modeling.	Non‐probability sample; response rate and full confounder set not detailed.
López‐Vallejo et al. (2025) [36]	Cross‐sectional	Cross‐Sectional Checklist	High Quality	Large, purpose‐built cancer survivorship cohort with a validated HRQoL instrument and fully adjusted models.	Response rate for START‐PR recruitment not detailed.
McSorley and Bacong (2025) [37]	Cross‐sectional secondary analysis	Cross‐Sectional Checklist	High Quality	Large, population‐representative, multi‐territory Behavioral Risk Factor Surveillance System sample following STROBE guidance.	Puerto Rico‐specific effect estimates not separately detailed from the pooled three‐territory estimate.
Yang, Li and An (2025) [38]	Quasi‐experimental repeated cross‐sections	Cross‐Sectional Checklist	Moderate‐High Quality	Quasi‐experimental matched difference‐in‐differences design strengthens causal interpretation relative to purely cross‐sectional studies in this review.	Based on repeated independent cross‐sections rather than a followed cohort; response rate not detailed.
Vázquez et al. (2026) [39]	Cross‐sectional comparative	Cross‐Sectional Checklist	Moderate Quality	Structured comparative design extending a well‐established cross‐national homelessness research program to a Puerto Rico site.	Puerto Rico‐specific sample size, response rate, and confounder adjustment not separately detailed.

## 4 Discussion

Chronic disease burden, socioeconomic disadvantage, acculturation, neighborhood and territorial context, and exposure to discrete structural shocks such as Hurricane Maria each showed independent and overlapping associations with self‐reported health across the 33 included studies. No single factor dominates the picture; the evidence is consistent with compounding influences that are specific to Puerto Rican populations and not always replicated in other Hispanic subgroups or in the general United States population.

### 4.1 Chronic Disease Burden and Physiological Stress

Across mainland and island samples, chronic disease burden, functional limitation, allostatic load, and depressive symptoms were the factors most consistently associated with poor self‐rated health. Castaneda‐Sceppa et al. (2010) [9] found that obesity, diabetes, depression, and cardiovascular history were each associated with poor physical function after adjustment for age, sex, education, and income. Todorova et al. (2013) [14] identified the same conditions as the factors most consistently associated with self‐rated health ratings in Boston‐area Puerto Rican adults, with acculturation moderating the strength of those associations. Mattei et al. (2010) [10] linked allostatic load to hypertension, diabetes, abdominal obesity, and cardiovascular disease, with association strengths comparable to or larger than those for metabolic syndrome across most outcomes, suggesting allostatic load may be a more comprehensive marker of cumulative disease burden than any single condition. Gago et al. (2024) [33], using the PROSPECT cohort, provided direct evidence that a single‐item self‐rated health indicator remains associated with medically diagnosed chronic disease status after adjustment for socioeconomic, demographic, and behavioral confounders, supporting self‐rated health's utility as a population‐level chronic disease indicator in a Puerto Rican sample specifically. Taken together, these findings are consistent with cumulative physiological stress from chronic social disadvantage, rather than any single clinical condition, as a central correlate of poor self‐reported health in this population; because the underlying studies are predominantly cross‐sectional, this pattern is best read as a consistent association rather than an established causal pathway (Section 5).

### 4.2 Acculturation and Measurement Validity

Acculturation showed a consistent but nuanced pattern. Greater English use and longer mainland residence were associated with more positive health self‐assessments in several studies, including Van Rompay et al. (2012) [12] and Todorova et al. (2013) [14]. Todorova et al. (2013) [14] also identified an important measurement concern: less acculturated individuals in objectively poor health tended to understate the severity of their condition, suggesting self‐rated health may conceal disparities in this population. Erving and Zajdel (2022) [25] extended this validity concern at the national level, finding that self‐rated health does not align consistently with morbidity patterns across ethnic groups, and that language of interview and years of United States residence partially account for these ethnic distinctions. These findings suggest that single‐item self‐rated health measures in less acculturated Puerto Rican patients may understate actual health burden, a possibility clinicians and researchers should weigh when interpreting such ratings.

### 4.3 Socioeconomic Barriers and Healthcare Access

Socioeconomic barriers operated at multiple levels. Poverty, low educational attainment, unemployment, and limited English proficiency were consistently associated with worse self‐rated health across study designs and geographic settings. Using 2019 Behavioral Risk Factor Surveillance System data, Frontera‐Escudero et al. (2023) [29] updated Puerto Rico's population‐level health‐related quality of life estimates for the first time since 2011, finding that poorer quality of life concentrated among older adults, lower‐income residents, and those with chronic conditions, immediately prior to the compounding effects of the COVID‐19 pandemic. Jimenez et al. (2015) [16] extended this socioeconomic framing by showing that relative economic disadvantage within a neighborhood, not just absolute poverty, was associated with elevated allostatic load over time. Jaen et al. (1998) [7] found that language barriers with providers and perceived distance to care were associated with higher rates of dysphoria in a predominantly Puerto Rican urban community; counterintuitively, individuals without insurance or a regular source of care were least likely to be dysphoric, a pattern the authors attributed to lower symptom recognition and help‐seeking among the most marginalized members of the sample. Calo et al. (2014) [15] identified unemployment and provider distrust as key correlates of perceived communication quality, linking socioeconomic position to how Puerto Ricans engage with and report on their healthcare interactions.

### 4.4 Nativity and Geographic Effects

Place of birth and current residence produced distinct health trajectories. Perez and Ailshire (2017) [18] found that older island‐residing Puerto Ricans were less likely to report poor self‐rated health, heart disease, stroke, and activity‐of‐daily‐living limitations than mainland‐residing older adults, a pattern the authors described as counterintuitive given the island's structural healthcare constraints; however, island residents carried higher rates of hypertension and diabetes, and island women had substantially worse outcomes than island men across most measures. This pattern, lower reported health burden alongside elevated chronic disease prevalence, is consistent with the measurement‐validity concern raised in Section 4.2: less acculturated island populations may underreport poor health, so self‐rated health measures could underestimate actual disease burden in this group. Sheftel and Heiland (2024) [34] found that birthplace, more than current residence, predicted disability prevalence in adulthood, pointing to early‐life socioeconomic conditions on the archipelago as a durable health exposure. Roy, Hughes and Yoshikawa (2013) [13] found that island‐born Puerto Ricans in ethnically dense, low‐socioeconomic‐status mainland neighborhoods reported the worst self‐rated health, a pattern not observed among mainland‐born peers, suggesting that isolation from resources compounds natal disadvantage. Extending this geographic lens beyond the mainland/island binary, McSorley and Bacong (2025) [37] found that residents of Puerto Rico and the other United States territories reported worse physical and mental health‐related quality of life than residents of the 50 states collectively in pooled 2021–2022 national surveillance data, underscoring both a substantive health disparity and a structural data gap: territories are routinely excluded from national health‐monitoring analyses, which limits the evidence base available for exactly the kind of disaggregated, territory‐specific research this review advocates.

### 4.5 Discrimination, Psychosocial Stress, and Coping

Discrimination and psychosocial stress contributed independently to self‐reported health outcomes. Woo et al. (2020) [21] found that perceived unfair treatment was associated with elevated depression risk, and that only four coping strategies, planning, acceptance, humor, and religion, moderated that association. Caraballo‐Cueto and Godreau [22] extended the discrimination construct beyond conventional ethno‐racial categories by incorporating a skin‐color scale into Puerto Rico's Behavioral Risk Factor Surveillance System, finding that darker‐skinned Puerto Ricans reported worse general health across both logistic regression and propensity‐score‐matched models, and that skin color outperformed standard federal race categories as a predictor of self‐reported health in this context. Lee‐Bravatti et al. [23] showed that a composite healthy lifestyle score was associated with measurable improvement in successful aging across physical and cognitive‐psychosocial domains over 2 years. McSorley, Tobin and Kuhn [31] identified a pattern unique to Puerto Ricans among the groups studied: lower internal political efficacy was associated with higher self‐rated health, the inverse of the pattern in Cuban, Mexican, and non‐Latino White groups, which the authors attributed to Puerto Rico's context of sustained political exclusion possibly decoupling political engagement from health perception.

### 4.6 Health Belief Model Application

The Health Belief Model was applied in this review as a post hoc organizing framework rather than as a hypothesis tested by the included studies or a criterion that determined study eligibility (Section 2.3); none of the 33 included studies were designed around the model, and most did not reference it directly. Applied in this interpretive capacity, the evidence maps onto its constructs in a way that is useful for structuring narrative synthesis, without implying that study authors intended or tested these constructs themselves. Perceived severity was reflected in findings where chronic disease burden, functional limitation, or physiological stress drove worse self‐rated health ratings. Perceived barriers appeared across studies as poverty, language, limited provider access, discrimination, and institutional distrust. Perceived benefits manifested in the protective associations of healthy behaviors, acculturation‐linked resources, social connectedness, and adaptive coping strategies. Self‐efficacy tracked with acculturation level, composite lifestyle scores, and behavioral resources for managing chronic conditions. Cues to action emerged from provider visits, communication quality, and culturally targeted engagement with the healthcare system. A subset of findings, most often those centered on demographic or structural modifiers such as race/ethnicity/country of origin or social support networks, did not map cleanly onto a single construct and are labeled as modifying factors in the coding table (Table 4) rather than force‐fit into a construct the original study did not measure. Read this way, the model highlights that many Puerto Rican individuals and communities face constraints across multiple constructs simultaneously, which is consistent with, though not proof of, the idea that single‐domain interventions are unlikely to be sufficient on their own.

### 4.7 Hispanic Disaggregation

Disaggregation from broader Hispanic or pan‐ethnic categories emerged as a recurring methodological theme across the included studies. Benjamins et al. [11], comparing Black, White, Mexican, and Puerto Rican respondents in a Chicago community health survey, found that Puerto Ricans reported lower self‐rated health than the other three groups even after adjustment for socioeconomic and behavioral covariates, an early empirical case for subgroup‐level rather than pan‐Hispanic or pan‐racial analysis. Lo et al. [20], drawing on 17 years of the National Health Interview Survey, found that the direction and strength of associations between social factors and self‐rated health differed meaningfully across Puerto Rican, Cuban, Mexican, Dominican, and Central and South American subgroups. Assari and Lankarani [17] similarly found that demographic and socioeconomic determinants of both physical and mental self‐rated health varied across 10 ethnic groups, with Puerto Ricans showing aging‐related patterns not seen among non‐Latino Whites. Together, these studies suggest that aggregating Puerto Ricans into a pan‐Hispanic category risks obscuring patterns specific to this population and producing policy or clinical recommendations that do not fit its actual health profile.

### 4.8 Cancer Survivorship and Disease‐Specific Quality of Life

Five studies in this review extend the evidence base to cancer survivorship and other disease‐specific populations. In the START‐PR cohort, López‐Vallejo et al. [36] found that cancer survivors in Puerto Rico with at least one comorbidity, most commonly hypertension, diabetes, depression, arthritis, or asthma, were more likely to report poor health‐related quality of life, with scores declining as comorbidity count increased; patient navigation services and perceived social support did not significantly modify this association, suggesting comorbidity management itself, rather than support‐service availability alone, may be the more direct lever for improving survivorship quality of life. At the national level, Reeve et al. [32] found that Cuban, Dominican, and Puerto Rican cancer survivors were more likely to fall into a “very low” health‐related quality of life cluster, roughly 15 points below United States population norms, a disparity that a pan‐Hispanic analytic approach would likely have obscured, reinforcing this review's broader disaggregation theme (Section 4.7). Santiago‐Pérez et al. [26] similarly found that 90 percent of gynecologic cancer patients in Puerto Rico reported at least one additional comorbidity, most often cardiovascular disease, which was associated with poorer quality‐of‐life items in adjusted models. Beyond cancer, Arce‐Ayala et al. [19] found that Puerto Rican patients with hereditary angioedema, a rare chronic condition, reported quality of life well below United States population norms across physical and mental component scores, and Lojo et al. [30] found that Puerto Ricans with inflammatory bowel disease and an ostomy reported higher quality of life the longer they had lived with the ostomy, with men reporting higher scores than women, a sex difference the authors suggested may reflect an opportunity for tarq2geted ostomy education. Across these disease‐specific studies, chronic comorbidity burden is a recurring correlate of poorer self‐reported and disease‐specific quality of life, consistent with the chronic‐disease pattern identified in Section 4.1 and extending it beyond general‐population samples to clinical populations.

### 4.9 Disaster Exposure, Social Connectedness, and Aging

A cluster of studies examines how discrete disaster exposure and social connectedness shape self‐reported health, particularly among older adults. Using a matched difference‐in‐differences design applied to 2016–2018 Behavioral Risk Factor Surveillance System data, Yang, Li and An (2025) [38] found statistically significant increases of 0.13 and 0.17 self‐reported poor mental and physical health days per month, respectively, following Hurricane Maria; because this design compares matched groups before and after a specific event rather than relying on a single cross‐sectional snapshot, it offers stronger quasi‐experimental support than most other studies in this review for a temporal link between a discrete structural exposure and self‐reported health decline. Two related studies examined protective factors among older adults roughly 2 years after the same hurricane: Buckley, Becker and Burnette [24] found that a validated measure of friend‐based social network ties was inversely associated with self‐rated health, with a significant indirect effect through psychological sense of community, while Buckley and Burnette [27], drawing on the same post‐disaster sample, found that psychological sense of community was positively associated with quality of life and that poorer self‐rated health and greater mental health symptoms were associated with lower quality of life. Kim et al. [35], surveying older adults again in 2021 during the COVID‐19 pandemic, found that both pandemic‐related attitudes and psychological sense of community were positively associated with quality of life, with sense of community partially mediating that relationship. Read together, these four studies suggest that psychological sense of community and social connectedness may function as a recurring protective resource for older Puerto Rican adults across at least two distinct disaster and public health emergency contexts.

### 4.10 Understudied and Intersecting Populations

A set of studies addresses populations that remain largely absent from the broader Puerto Rican self‐rated health literature. Esteban et al. [28], the first empirical Puerto Rico‐based study of this population identified in this review, documented experiences of discrimination and violence among intersex‐identifying adults and examined differences in quality of life and psychosocial well‐being relative to an endosex comparison group; given the pilot sample size (*n* = 12 intersex participants), these findings are best read as descriptive and hypothesis‐generating rather than conclusive, but they establish a needed empirical foothold for future research. Vázquez et al. [39] extended a cross‐national comparative research program on homelessness to include Puerto Rico for the first time, finding elevated chronic illness, disability, and poor perceived health among women experiencing homelessness on the island, within a broader pattern in which economic development level only partially explained cross‐national differences in health status among homeless women in Nicaragua, Argentina, Puerto Rico, and Spain. Rao et al. [8], comparing English‐ and Spanish‐speaking adults living with HIV/AIDS in the continental United States and Puerto Rico, found that health‐related quality of life differed by language of survey administration and clinical site, underscoring the need for language‐ and culture‐concordant outcome measurement in HIV care specifically, and, more broadly, for self‐reported health research generally. Together, these three studies illustrate that structural marginalization, whether tied to gender identity and sex characteristics, housing status, or a stigmatized chronic infectious disease, compounds with the socioeconomic and nativity‐related disadvantages documented in Sections 4.1 through 4.4 to shape self‐reported health among Puerto Ricans who occupy multiple marginalized positions simultaneously.

## 5 Limitations

Several methodological constraints bound the scope and generalizability of this review. As a single‐author study, all stages of the review process, including database searching, deduplication, title and abstract screening, full‐text eligibility decisions, data extraction, Health Belief Model coding, and quality appraisal, were completed by one person without independent verification. Single‐author reviews carry an elevated risk of selection bias, as there is no second screener to challenge or confirm eligibility and coding decisions. No formal inter‐rater reliability was established; two iterative rounds of both Health Belief Model coding and Joanna Briggs Institute critical appraisal were completed to improve internal consistency, but independent verification was not performed.

Nine databases were searched (MEDLINE, Academic Search Ultimate, CINAHL, APA PsycInfo, Education Research Complete, Health and Psychosocial Instruments, Political Science Complete, America: History & Life, and ERIC). Even with this expansion, institutional access did not extend to Scopus, Web of Science, or Embase, which may index additional eligible studies not captured here. Grey literature sources, including dissertations, conference proceedings, and government or institutional reports, were not systematically searched; while this scope decision kept the review focused on peer‐reviewed empirical literature, it may have excluded relevant findings not published in indexed journals. The English‐language restriction likely omitted studies published in Spanish‐language journals, which may contain important findings from Puerto Rico‐based researchers with direct community access. The 30‐year search window (1996‐2026) may also have missed earlier studies that established baseline health patterns for this population.

Twenty‐nine of the 33 included studies used cross‐sectional or quasi‐experimental repeated‐cross‐section designs, which limit the strength of causal claims about the relationships summarized in Sections 4.1 through 4.5 and 4.8 through 4.10. Directionality between self‐rated health and its correlates, including depressive symptoms, allostatic load, and acculturation, generally cannot be established from these data; the matched difference‐in‐differences design used by Yang, Li and An [38] (Section 4.9) is a partial exception that strengthens confidence in a temporal link between Hurricane Maria and self‐reported health decline specific ston Puerto Rican Health Study and one on a Puerto Rico‐based inflammatory bowel disease registry, represent the most robust longitudinal or disease‐specific resources currently available for this population, but their geographic and clinical concentration limits generalizability to the broader mainland diaspora and to Puerto Rican communities not represented in these specific cohorts.

Self‐rated health and related outcomes were operationalized inconsistently across studies. Some used single‐item global ratings; others used health‐related quality of life scales, disability measures, disease‐specific instruments, or composite indices. This measurement heterogeneity precluded meta‐analysis and made direct comparison of effect sizes across studies unreliable. The finding by Todorova et al. [14] that less acculturated individuals may systematically underreport poor health (Section 4.2) introduces additional measurement uncertainty that could affect any study in this review that did not control for language of interview or acculturation level; Gago et al. [33] provides some reassurance on this point by directly validating single‐item self‐rated health against medically diagnosed chronic disease status in a Puerto Rico‐based sample, but this validation has not been replicated across the full range of instruments used in this review.

Subgroup variation within the Puerto Rican population, including by sex, age, nativity, generation status, sexual and gender minority status, and housing status, was not consistently reported across studies, limiting the ability to draw conclusions for specific subpopulations. Women, older adults, island‐born individuals, and the newly identified subpopulations discussed in Section 4.10, intersex‐identifying adults and women experiencing homelessness, emerged as vulnerable subgroups in the available evidence, but sample sizes within these strata were frequently small and insufficient for definitive inference. Publication bias toward statistically significant findings may further skew the available evidence base.

These constraints bear directly on how the findings in Section Conclusion, 4 should be read. The cross‐sectional predominance described above means the chronic‐disease and socioeconomic associations summarized in Sections 4.1 and 4.3 are best interpreted as consistent correlates rather than established causes. The acculturation‐related measurement bias documented in Section 4.2 suggests that self‐rated health estimates for less acculturated subgroups, including many island residents discussed in Section 4.4, may understate true disease burden, which in turn tempers the apparently favorable self‐rated health findings for island‐residing older adults reported by Perez and Ailshire [18]. Finally, the geographic concentration of the strongest longitudinal evidence in the Boston Puerto Rican Health Study means that findings described as robust in Sections 4.1, and 4.3 carry more certainty for that specific mainland community than for Puerto Rican populations elsewhere, including the island‐based, disaster‐affected, and structurally marginalized subpopulations discussed in Sections 4.4, and 4.10.

## 6 Conclusion

Poor self‐reported health among Puerto Ricans appears to reflect the intersection of chronic disease burden, socioeconomic disadvantage, structural barriers to care, and the lasting effects of early‐life conditions, rather than any single modifiable factor. Acculturation shapes not only health outcomes but also how health is perceived and reported, meaning that standard self‐rated health measures may underestimate disparities among less acculturated individuals. Island birth is associated with durable health differences that persist across geographic transitions, and current residence alone does not appear to account for that association. This evidence extends to cancer survivorship, disaster exposure, social connectedness in later life, and structurally marginalized subpopulations, while reinforcing the review's central methodological finding: disaggregating Puerto Rican populations from pan‐Hispanic categories is important for producing research and clinical data that reflect this population's actual health profile, rather than assuming Puerto Ricans' experience mirrors that of other Hispanic or Latino subgroups.

Future research would benefit from expanded longitudinal study of self‐rated health in Puerto Rican populations. The Boston Puerto Rican Health Study remains the field's most methodologically rigorous cohort resource and has produced the majority of longitudinal evidence reviewed here; replicating its design in other Puerto Rican communities, on the mainland and the archipelago, and extending comparable cohort methods to disease‐specific and disaster‐affected populations, could strengthen statistical power and geographic generalizability. Given the quasi‐experimental evidence that Hurricane Maria was associated with measurable self‐reported health decline (Section 4.9) [38], continued longitudinal attention to disaster exposure and recovery, alongside the structural data gaps affecting Puerto Rico and other United States territories identified by McSorley and Bacong [37], appears warranted. Culturally validated self‐reported health measures that account for acculturation level, together with provider education on Puerto Rican health context, are quality‐of‐care considerations that this evidence base suggests deserve more direct and sustained attention, alongside continued research attention to intersecting and understudied subpopulations, including intersex‐identifying adults, women experiencing homelessness, and people living with HIV/AIDS, identified in this updated synthesis.

## Author Contributions

**Anthony Vincent Razzano:** conceptualization, writing – review and editing, methodology, writing – original draft, formal analysis, data curation. All authors have read and approved the final version of the manuscript.

## Funding

The author has nothing to report.

## Ethics Statement

Ethical approval was not required for this systematic review, as it analyzes previously published data and does not involve direct human participants.

## Conflicts of Interest

The author declares no conflicts of interest.

## Transparency Statement

Anthony Vincent Razzano affirms that this manuscript is an honest, accurate, and transparent account of the study being reported; that no important aspects of the study have been omitted; and that any discrepancies from the study as planned (and, as relevant, registered) have been explained.

## Data Availability

Data sharing is not applicable to this article, as no datasets were generated or analyzed beyond the published studies cited herein. All included studies are cited in the reference list and are publicly available through their respective sources.

## References

[1] M. Prybylo , S. Miller , and T. Kelechi , Evaluation of Self‐Reported Health as a Tool for Identifying Risk of Hospitalization and Emergency Department Use (Population Health Management, 2021). 10.1016/j.gerinurse.2025.103659 41172652

[2] I. Lafarga Previdi and C. M. Vélez Vega , “Health Disparities Research Framework Adaptation to Reflect Puerto Rico's Socio‐Cultural Context,” International Journal of Environmental Research and Public Health 17, no. 22 (November 2020): 8544. 33217956 10.3390/ijerph17228544 PMC7698747

[3] K. L. Tucker , J. Mattei , S. E. Noel , et al., “The Boston Puerto Rican Health Study, a Longitudinal Cohort Study on Health Disparities in Puerto Rican Adults: Challenges and Opportunities,” BMC Public Health 10, no. 1 (March 2010): 107. 20193082 10.1186/1471-2458-10-107 PMC2848197

[4] Z. Munn , C. Stern , E. Aromataris , C. Lockwood , and Z. Jordan , “What Kind of Systematic Review Should I Conduct? A Proposed Typology and Guidance for Systematic Reviewers in the Medical and Health Sciences,” BMC Medical Research Methodology [Internet] 18, no. 1 (January 2018): 1–9, https://pmc.ncbi.nlm.nih.gov/articles/PMC5761190/. 29316881 10.1186/s12874-017-0468-4 PMC5761190

[5] M. Page , J. McKenzie , P. Bossuyt , et al., “The PRISMA 2020 Statement: An Updated Guideline for Reporting Systematic Reviews,” British Medical Journal [Internet] 372, no. 71 (2021): 1–5, https://www.bmj.com/content/372/bmj.n71. 10.1136/bmj.n71 PMC8005924 33782057

[6] A. Pormehr‐Yabandeh , T. Aghamolaei , Z. Hosseini , N. Roozbeh , and A. Ghanbarnezhad , “Application of the Health Belief Model to Explain Pre‐Conception Care Among Women With Sickle Cell Disease: A Cross‐Sectional Study,” BMC Pregnancy and Childbirth 25, no. 1 (September 2025): 922. 40898065 10.1186/s12884-025-08070-5 PMC12406346

[7] C. R. Jaén , S. Chadha , L. M. Tumiel , et al., “Patterns of Dysphoria in a Puerto Rican Urban Community,” Journal of the National Medical Association 90, no. 2 (1998): 93–98. 9510623 PMC2608315

[8] D. Rao , E. A. Hahn , D. Cella , and L. Hernandez , “The Health Related Quality of Life Outcomes of English and Spanish Speaking Persons Living With HIV/AIDS From the Continental United States and Puerto Rico,” AIDS Patient Care and STDs 21, no. 5 (2007): 339–346. 17518526 10.1089/apc.2006.0124

[9] C. Castaneda‐Sceppa , L. L. Price , S. E. Noel , J. Bassett Midle , L. M. Falcon , and K. L. Tucker , “Physical Function and Health Status in Aging Puerto Rican Adults: The Boston Puerto Rican Health Study,” Journal of Aging and Health 22, no. 5 (May 2010): 653–672. 20495158 10.1177/0898264310366738 PMC4413929

[10] J. Mattei , S. Demissie , L. M. Falcon , J. M. Ordovas , and K. Tucker , “Allostatic Load Is Associated With Chronic Conditions in the Boston Puerto Rican Health Study,” Social Science & Medicine (1982) 70, no. 12 (June 2010): 1988–1996. 20381934 10.1016/j.socscimed.2010.02.024 PMC2907654

[11] M. R. Benjamins , J. Hirschman , J. Hirschtick , and S. Whitman , “Exploring Differences in Self‐Rated Health Among Blacks, Whites, Mexicans, and Puerto Ricans,” Ethnicity & Health 17, no. 5 (2012): 463–476. 22288772 10.1080/13557858.2012.654769

[12] M. I. Van Rompay , N. M. McKeown , C. Castaneda‐Sceppa , L. M. Falcón , J. M. Ordovás , and K. L. Tucker , “Acculturation and Sociocultural Influences on Dietary Intake and Health Status Among Puerto Rican Adults in Massachusetts,” Journal of the Academy of Nutrition and Dietetics 112, no. 1 (January 2012): 64–74. 22389874 10.1016/j.jada.2011.08.049 PMC3289968

[13] A. L. Roy , D. Hughes , and H. Yoshikawa , “Intersections Between Nativity, Ethnic Density, and Neighborhood SES: Using An Ethnic Enclave Framework to Explore Variation in Puerto Ricans’ Physical Health,” American Journal of Community Psychology 51, no. 3–4 (2013 Jun): 468–479. 23314837 10.1007/s10464-012-9564-0

[14] I. L. Todorova , K. L. Tucker , M. P. Jimenez , A. K. Lincoln , S. Arevalo , and L. M. Falcón , “Determinants of Self‐Rated Health and the Role of Acculturation: Implications for Health Inequalities,” Ethnicity & Health 18, no. 6 (2013 Feb 21): 563–585. 23425383 10.1080/13557858.2013.771147 PMC3758374

[15] W. A. Calo , A. P. Ortiz , V. Colon‐Lopez , S. Krasny , and G. Tortolero‐Luna , “Factors Associated With Perceived Patient‐Provider Communication Quality Among Puerto Ricans,” Journal of Health Care for the Poor and Underserved 25, no. 2 (2014): 491–502. 24858864 10.1353/hpu.2014.0074 PMC4154245

[16] M. P. Jiménez , T. L. Osypuk , S. Arevalo , K. L. Tucker , and L. M. Falcon , “Neighborhood Socioeconomic Context and Change in Allostatic Load Among Older Puerto Ricans: The Boston Puerto Rican Health Study,” Health & Place 33 (May 2015): 1–8. 25706323 10.1016/j.healthplace.2015.02.001 PMC4409499

[17] M. Moghani Lankarani and S. Assari , “Demographic and Socioeconomic Determinants of Physical and Mental Self‐Rated Health Across 10 Ethnic Groups in the United States,” International Journal of Epidemiologic Research 4, no. 3 (2017): 185–193. 10.15171/ijer.2017.02 PMC6703549 31435528

[18] C. Pérez and J. A. Ailshire , “Aging in Puerto Rico: A Comparison of Health Status Among Island Puerto Rican and Mainland U.S. Older Adults,” Journal of Aging and Health 29, no. 6 (June 2017): 1056–1078. 28599584 10.1177/0898264317714144 PMC5746478

[19] Y. M. Arce‐Ayala , Y. Diaz‐Algorri , T. Craig , and C. Ramos‐Romey , “Clinical Profile and Quality of Life of Puerto Ricans With Hereditary Angioedema,” Allergy and Asthma Proceedings 40, no. 2 (March 2019): 103–110. 30819279 10.2500/aap.2019.40.4200

[20] C. C. Lo , J. L. Adame , and T. C. Cheng , “Explaining Chronic Illness and Self‐Rated Health Among Immigrants of Five Hispanic Ethnicities,” Journal of Racial and Ethnic Health Disparities 7, no. 1 (2020): 177–191. 31654338 10.1007/s40615-019-00647-z

[21] B. Woo , K. Wang , and L. M. Falcón , “Unfair Treatment, Coping Strategies, and Depression Among Puerto Ricans in Boston,” Cultural Diversity & Ethnic Minority Psychology 26, no. 2 (April 2020): 229–238. 31021145 10.1037/cdp0000291

[22] J. Caraballo‐Cueto and I. P. Godreau , “Colorism and Health Disparities in Home Countries: The Case of Puerto Rico,” Journal of Immigrant and Minority Health 23, no. 5 (October 2021): 926–935. 34097164 10.1007/s10903-021-01222-7

[23] M. A. Lee‐Bravatti , H. J. O'neill , R. C. Wurth , et al., “Lifestyle Behavioral Factors and Integrative Successful Aging Among Puerto Ricans Living in the Mainland United States,” Journals of Gerontology. Series A, Biological Sciences and Medical Sciences 76, no. 6 (May 2021): 1108–1116. 33045072 10.1093/gerona/glaa259 PMC8248899

[24] T. D. Buckley , T. D. Becker , and D. Burnette , “Validation of the Abbreviated Lubben Social Network Scale (LSNS‐6) and Its Association With Self‐Rated Health Amongst Older Adults in Puerto Rico,” Health & Social Care in the Community 30, no. 6 (November 2022): 5527. 10.1111/hsc.13977 36039906

[25] C. L. Erving and R. Zajdel , “Assessing the Validity of Self‐Rated Health Across Ethnic Groups: Implications for Health Disparities Research,” Journal of Racial and Ethnic Health Disparities 9, no. 2 (April 2022): 462–477. 33544329 10.1007/s40615-021-00977-x

[26] G. G. Santiago‐Pérez , C. P. Amaya‐Ardila , S. A. Umpierre , and A. P. Ortiz‐Martinez , “Effect of Chronic Comorbidities on Quality of Life of Gynecologic Cancer Patients in Puerto Rico,” Revista Panamericana de Salud Pública 46 (April 2022): e29. 35432504 10.26633/RPSP.2022.29 PMC9004689

[27] T. D. Buckley and D. Burnette , “Psychological Sense of Community, Self‐Rated Health and Quality of Life Among Older Adults in Puerto Rico Two Years After Hurricane María,” Journal of Gerontological Social Work 66, no. 4 (2023): 512–529. 36217794 10.1080/01634372.2022.2133200

[28] C. Esteban , D. I. Ortiz‐Rodz , Y. I. Muñiz‐Pérez , et al., “Quality of Life and Psychosocial Well‐Being Among Intersex‐Identifying Individuals in Puerto Rico: An Exploratory Study,” International Journal of Environmental Research and Public Health 20, no. 4 (February 2023): 2899. 36833596 10.3390/ijerph20042899 PMC9957316

[29] I. Frontera‐Escudero , J. A. Bartolomei , A. Rodríguez‐Putnam , and L. Claudio , “Sociodemographic and Health Risk Factors Associated With Health‐Related Quality of Life Among Adults Living in Puerto Rico in 2019: A Cross‐Sectional Study,” BMC Public Health 23, no. 1 (November 2023): 2150. 37924064 10.1186/s12889-023-17115-3 PMC10623836

[30] J. J. Lojo , R. de la Villa , M. M. Vega‐Torres , and E. A. Torres , “Ostomy‐Related Quality of Life in Puerto Ricans Living With Inflammatory Bowel Disease: A Prospective Cohort Study,” Journal of wound, ostomy, and continence nursing: official publication of The Wound, Ostomy and Continence Nurses Society 50, no. 3 (May‐Jun 2023): 222–226. 37146114 10.1097/WON.0000000000000964

[31] A. M. M. McSorley , C. S. Thomas Tobin , and R. Kuhn , “The Relationship Between Political Efficacy and Self‐Rated Health: An Analysis of Mexican, Puerto Rican, and Cuban Subgroups Compared to Non‐Latinx Whites in the United States,” SSM ‐ Population Health 22 (March 2023): 101390. 37251508 10.1016/j.ssmph.2023.101390 PMC10214832

[32] B. B. Reeve , K. D. Graves , L. Lin , et al., “Health‐Related Quality of Life by Race, Ethnicity, and Country of Origin Among Cancer Survivors,” JNCI: Journal of the National Cancer Institute 115, no. 3 (March 2023): 258–267. 36519827 10.1093/jnci/djac230 PMC9996211

[33] C. Gago , H. J. O'neill , M. Tamez , A. López‐Cepero , J. F. Rodríguez‐Orengo , and J. Mattei , “Self‐Rated Health and Medically Diagnosed Chronic Disease Association Among Adults in Puerto Rico,” Ethnicity & Disease 33, no. 4 (April 2023): 140–149. 38854413 10.18865/ed.33.4.140 PMC11155621

[34] M. G. Sheftel and F. W. Heiland , “The Role of Place of Birth and Residence in Puerto Rican Health Disparities: Evidence From Disability Prevalence Among Archipelago‐ vs. Mainland‐Born Puerto Ricans,” Journal of Aging and Health 36, no. 1–2 (January 2024): 67–84. 37115825 10.1177/08982643231172643 PMC10693743

[35] S. Kim , T. D. Becker , M. R. Morgan , and D. Burnette , “COVID‐19 Attitudes and Quality of Life in Older Adults in Puerto Rico: The Mediating Role of Psychological Sense of Community,” Journal of Applied Gerontology 44 (2025): 1068–1077, 10.1177/07334648241301482. 39654340

[36] D. López‐Vallejo , C. M. Pérez , L. González‐Sepúlveda , and M. Soto‐Salgado , “Examining Health‐Related Quality of Life in Cancer Survivors: Cross‐Sectional Associations With Comorbidities, Navigation Services Use, and Perceived Social Support,” Cancers 17, no. 23 (2025): 3784. 41374986 10.3390/cancers17233784 PMC12691021

[37] A. M. M. McSorley and A. M. Bacong , “Health‐Related Quality of Life in the US Territories of Puerto Rico, Guam, and the Virgin Islands,” JAMA Network Open 8, no. 4 (2025 Apr 17): e255646. 40244588 10.1001/jamanetworkopen.2025.5646 PMC12006872

[38] Y. Yang , D. Li , and R. An , “Impact of Hurricane Maria on Mental and Physical Health Among Puerto Ricans: A Matched Difference‐in‐Differences Analysis,” Journal Of Human Behavior In The Social Environment (2025): 1–15, 10.1080/10911359.2025.2563004.

[39] J. J. Vázquez , S. Torrego , M. Lenta , J. Di Iorio , A. Suarez , and S. Panadero , “Health Status of Women Experiencing Homelessness Across Diverse Economic and Cultural Contexts: A Comparative Analysis in Nicaragua, Argentina, Puerto Rico, and Spain,” Journal of Health Care for the Poor and Underserved 37 (2026): 493–508, 10.1353/hpu.2026.a992126. 42290348

